# Indole‐3‐Propionic Acid Alleviates Metabolic Dysfunction‐Associated Fatty Liver Disease via AHR/AMPK Signaling Activation

**DOI:** 10.1002/fsn3.72206

**Published:** 2026-08-07

**Authors:** Yanting Huang, Meimei Yu, Jiaxin Lu, Chujun Lin, Shuhuan Li, Yalin Chen, Xin Chen, Hongliang Xue, Jing Chen, Lijie Su

**Affiliations:** ^1^ Department of Nutrition and Food Hygiene, School of Public Health Guangzhou Medical University Guangzhou China; ^2^ The Key Laboratory of Advanced Interdisciplinary Studies, the First Afffliated Hospital Guangzhou Medical University Guangzhou China; ^3^ Sino‐French Hoffmann Institute, School of Basic Medical Science Guangzhou Medical University Guangzhou China

**Keywords:** AHR nuclear translocation, AMPK phosphorylation, hepatic steatosis, lipid deposition, lipid synthesis

## Abstract

Metabolic dysfunction‐associated fatty liver disease (MAFLD) is a health problem worthy of attention worldwide, characterized by excessive lipid accumulation in the liver. Previous studies have shown that indole‐3‐propionic acid (IPA) can ameliorate MAFLD; however, the regulatory mechanism by which IPA affects lipid synthesis requires further investigation. In vitro, FFA‐induced HepG2 cells were treated with IPA (15–35 μM), and hepatic lipid accumulation was evaluated by Oil Red O staining. RNA‐seq and Western blotting were employed to analyze AHR/AMPK signaling activation, and pharmacological inhibitors and agonists targeting AHR and AMPK were applied to explore the regulatory mechanism of IPA on lipid deposition. In vivo, C57BL/6J mice on a high‐fat diet received 50 mg/kg IPA for 16 weeks, followed by histopathological evaluation and assessment of AHR/AMPK pathway activation. Results showed that IPA dose‐dependently reduced lipid deposition in HepG2 cells. IPA inhibited lipid synthesis by activating AHR (inducing CYP1A1 expression and nuclear translocation) and promoting AMPK phosphorylation, thereby downregulating SREBP‐1c and FAS expression. Pharmacological experiments demonstrated that IPA‐induced AMPK activation was dependent on AHR nuclear translocation. Moreover, phosphorylated AMPK further promoted AHR nuclear translocation, indicating the existence of a potential bidirectional crosstalk between AHR and AMPK. In animal studies, IPA mitigated HFD‐induced hepatic steatosis through activation of the AHR/AMPK signaling pathways. In conclusion, indole‐3‐propionic acid may alleviate MAFLD by ameliorating high‐fat diet‐induced hepatic lipid deposition through AHR/AMPK signaling activation.

## Graphical abstract

IPA promotes AHR nuclear translocation and AMPK phosphorylation. Phosphorylated AMPK may in turn facilitate AHR translocation, suggesting a potential reciprocal interplay that contributes to alleviating hepatic lipid deposition in MAFLD.

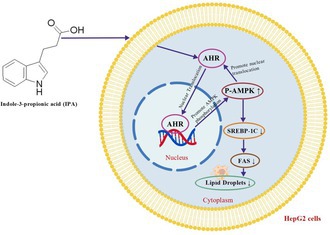

**Abbreviations**
AHR: aryl hydrocarbon receptor
AHRint: AHR inhibitor (AHR antagonist 5 free base)
AKN: AMPK agonist (Ampkinone)
ALT: alanine aminotransferase
AMPK: AMP‐activated protein kinase
AMPKint: AMPK inhibitor (AMPK‐IN‐3)
ANOVA: one‐way analysis of variance
AST: aspartate aminotransferase
CYP1A1: cytochrome P450 family 1 subfamily A member 1
DEGs: differentially expressed genes
ELISA: enzyme‐linked immunosorbent assay
FAS: fatty acid synthase
FFA: free fatty acid(s)
HFD: high‐fat diet
I3A: indole‐3‐acetic acid
Il‐1β: Interleukin‐1 beta
Il‐6: Interleukin‐6
IPA: indole‐3‐propionic acid
KA: kynurenic acid
LFD: low‐fat diet
MAFLD: Metabolic dysfunction‐associated fatty liver disease
MASH: Metabolic dysfunction‐associated steatohepatitis
NAFLD: nonalcoholic fatty liver disease
SREBP‐1c: sterol regulatory element‐binding protein 1c
TC: total cholesterol
TG: triglycerides
Tnf‐α: Tumor necrosis factor‐alpha
WB: western blotting

## 1 Introduction

Metabolic dysfunction‐associated fatty liver disease (MAFLD) is a fatty liver disorder caused by systemic metabolic dysregulation. In 2020, a global panel of experts suggested changing the name of nonalcoholic fatty liver disease (NAFLD) to MAFLD (Gofton et al. 2022). As the most prevalent chronic liver disease, MAFLD carries a risk of progression from initial lipid accumulation to steatohepatitis (MASH), fibrosis, and eventual cirrhosis (Rinella et al. 2023). Globally, the prevalence of MAFLD is approximately 25% (Bessone et al. 2019), a figure that continues to rise, particularly among individuals with obesity and diabetes (Badmus et al. 2022). Up to one‐third of patients with MAFLD may develop progressive steatohepatitis and fibrosis, which can ultimately lead to cirrhosis, hepatocellular carcinoma, and death (Simon et al. 2021; Vernon et al. 2011). The hallmark features of MAFLD encompass abnormal lipid deposition in the liver (Carotti et al. 2020), coupled with inflammation and insulin resistance (Younossi et al. 2018). In MAFLD, excessive hepatic lipid accumulation promotes the production of lipotoxic substances, leading to mitochondrial dysfunction (Zheng et al. 2023), endoplasmic reticulum stress, and excessive generation of reactive oxygen species (ROS) (Di Ciaula et al. 2021). Therefore, reducing the accumulation of fat in the liver is a promising method for the prevention and treatment of MAFLD.

The human gut harbors trillions of microorganisms, predominantly symbiotic bacteria, which are collectively referred to as the gut microbiota (Marano et al. 2025). The gut microbiome is one of the key factors influencing human health, and its functional impacts involve individual development, immune regulation, and metabolism (Ullah et al. 2025). Studies have demonstrated that gut microbiota‐derived indole metabolites contribute to the prevention and treatment of MAFLD (Alghamdi et al. 2024). Indole‐3‐propionic acid (IPA), a tryptophan‐derived gut bacterial metabolite, has been demonstrated to reduce inflammation via the NF‐κB pathway (Zhao et al. 2019) and to exhibit protective effects against liver fibrosis (Luo et al. 2025). Studies have shown that another tryptophan metabolite, indole‐3‐acetic acid (I3A), inhibits lipogenesis in vitro. I3A treatment significantly downregulated the expression of lipogenesis‐related genes SREBP1c and FAS in murine hepatocytes (Krishnan et al. 2019). Given the structural similarity between IPA and I3A, it is plausible that they share similar functions. This suggests that IPA might also possess the ability to attenuate hepatic lipid synthesis. Therefore, this study investigated the effects of IPA on hepatic lipogenesis.

The aryl hydrocarbon receptor (AHR), a bHLH‐PAS transcription factor, is characterized by its ligand‐dependent activation (Li, Innocentin, et al. 2011). Emerging evidence has demonstrated that AHR not only participates in xenobiotic metabolism but also regulates diverse endogenous physiological processes, including cell proliferation, differentiation, immune responses, oxidative stress, and tissue homeostasis (Huang et al. 2023; Mulero‐Navarro and Fernandez‐Salguero 2016; Perepechaeva and Grishanova 2020). AHR activation depends on ligand binding, with ligands originating from either exogenous sources (e.g., environmental contaminants) or endogenous metabolites (e.g., tryptophan derivatives). Upon activation, AHR translocates to the nucleus, subsequently dimerizes with ARNT, and ultimately binds to specific genomic sequences known as AHR‐responsive elements (AhREs) to regulate downstream gene expression (Panda et al. 2023). AHR activation in HFD‐fed mice leads to the downregulation of genes critical for lipid and cholesterol homeostasis, such as FASN and SREBP‐1c (Huang 2024). Emerging evidence positions AHR agonists as key regulators of metabolic disease, orchestrating a multi‐organ circuitry that integrates immune responses and gut microbial metabolism to alter disease trajectory (Gourronc et al. 2020; Lin et al. 2019; Natividad et al. 2018). Studies have shown that tryptophan‐derived indole metabolites have been identified as ligands for the aryl hydrocarbon receptor (AHR) (Hyun 2021). Consequently, the interplay between gut microbiota, tryptophan‐derived indole metabolites, and AHR may exert significant influence on the development and progression of MAFLD (Xu et al. 2021). IPA has been identified as a non‐canonical AHR ligand (Zhang et al. 2022). Therefore, whether IPA influences lipid synthesis through AHR, this issue needs to be further explored in this study.

Amp‐activated protein kinase (AMPK), an important metabolic controller, modulates the expression and activity of multiple lipid‐metabolizing enzymes and transcription factors to maintain lipid homeostasis (Steinberg and Hardie 2023). Sterol regulatory element‐binding protein 1c, one of the numerous regulatory targets of AMPK, is the primary transcriptional regulator of hepatic lipogenic genes (Geng and Guo 2024). It controls the production of important enzymes involving fatty acids synthesis, including fatty acid synthase (FAS) (Feng et al. 2025; Harley et al. 2022). AMPK directly phosphorylates SREBP‐1c, thereby inhibiting its activity and function, and plays a pivotal role in regulating lipid metabolism and fatty acid synthesis (Han et al. 2019). Studies have demonstrated that the AMPK signaling pathway plays a crucial role in ameliorating lipid metabolism disorders (Fang et al. 2022), thereby highlighting its potential as a therapeutic target for MAFLD. Therefore, this study examined whether IPA can activate the AMPK pathway, thereby suppressing hepatic lipid accumulation.

Recent studies have revealed that the tryptophan metabolite indole‐3‐carboxaldehyde (I3A) co‐activates both the AHR and AMPK signaling pathways, thereby enhancing intestinal barrier function and ameliorating colitis (Shi et al. 2025). Similarly, in the liver, the endogenous tryptophan metabolite kynurenic acid (KA) activates AMPK in an AHR‐dependent manner, effectively suppressing hepatocyte lipogenesis (Patil et al. 2025). Existing studies have indicated that AHR may mediate the activation of AMPK. However, whether AMPK reciprocally influences AHR remains unclear. These findings raise a deeper question: does a more intimate, bidirectional feedback dialogue mechanism exist between AHR and AMPK? In particular, whether IPA, as a natural ligand, can utilize and reinforce this AHR/AMPK axis remains poorly understood. Therefore, this study aimed to investigate the protective role of IPA against MAFLD and to specifically elucidate whether its effects were mediated through the activation of the AHR/AMPK signaling axis.

## 2 Materials and Methods

### 2.1 Animals and Treatment

Specific‐pathogen‐free male C57BL/6J mice were used in this study. These mice were raised in the experimental animal center of Guangzhou Medical University. This study was conducted in accordance with protocols approved by the Animal Ethics Committee of Guangzhou Medical University (Approval No. GY2022‐084). Mice adapted to the environment for 7 days, and then were divided into three groups: control group (LFD, *n* = 5), model group (HFD, *n* = 5) and indole propionic acid intervention group (HFD + IPA, *n* = 5). A total of 15 animals were used in the study. The LFD group received D12450H feed, while the other two groups maintained D12451 high‐fat feed. Indole‐3‐propionic acid (IPA) was administered once daily by gavage at the dosage 50 mg per kilogram of body weight. The dosage of the intervention was selected according to our preliminary studies as well as established literature (Lu et al. 2025). The intervention lasted for 16 weeks. At week 15, after a 12‐h fast, fasting blood glucose was measured. Glucose was then administered by oral gavage at 1 g/kg body weight. Blood glucose levels were determined at 0, 15, 30, 60, and 120 min after glucose administration, and the area under the curve (AUC) was calculated. At the end of the 16‐week experiment, animals were anesthetized by intraperitoneal injection of pentobarbital at a dose of 50 mg/kg body weight. Then, blood samples were collected and stored at room temperature for 30 min. After solidification, the sample was centrifuged for 15 min (4°C, 3000 × g). The obtained serum should be stored in the refrigerator at −80°C immediately. At the same time, liver and adipose tissues were collected, processed, and snap‐frozen in cryovials at −80°C for long‐term storage. Detailed information of all reagents and antibodies used in this study is provided in Table S1.

### 2.2 Biochemical Analysis

The concentrations of total cholesterol (TC) and triglycerides (TG) were quantified using commercial assay kits (Nanjing Jiancheng Bioengineering Institute). Additionally, serum alanine aminotransferase (ALT) and aspartate aminotransferase (AST) levels were determined with commercially available ELISA kits (Jiangsu Enzyme Immunoassay Industrial Co. Ltd. for TC/TG; Jiangsu Yutong Biotechnology Co. Ltd. for ALT/AST). All measurements were performed strictly according to the manufacturers' protocols.

### 2.3 Histopathology and Oil Red O Staining Analysis

Following fixation in 4% paraformaldehyde, liver tissues were dehydrated, embedded in paraffin, and subjected to H&E staining after sectioning. Alternatively, for lipid visualization, tissues were embedded in OCT compound, and cryosections were subjected to Oil Red O staining. The lipid‐positive areas were subsequently quantified using ImageJ software.

### 2.4 Cell Culture and Treatment

HepG2 cells (Cell Bank of the Academy of Sciences) were cultured in complete MEM under standard conditions (37°C, 5% CO_2_) and were treated according to experimental protocols upon attaining 80% confluence.

HepG2 cells were incubated for 36 h in complete culture medium containing a free fatty acid (FFA) mixture complexed with 5% fatty acid‐free bovine serum albumin (BSA) to induce steatosis. The FFA mixture comprised oleic acid (OA) and palmitic acid (PA) at a 2:1 M ratio (0.67 mM OA and 0.33 mM PA; total FFA concentration: 1 mM). The complete culture medium was supplemented with 10% fetal bovine serum (FBS) and 1% (v/v) penicillin–streptomycin.

IPA was initially dissolved in anhydrous ethanol, diluted with sterile water to prepare a stock solution, and further diluted in complete culture medium to the final working concentration (final ethanol concentration < 0.1%). Control cells were treated with an equivalent volume of BSA or vehicle in the absence of FFA or IPA.

To evaluate the effects of IPA on lipid deposition, cells were divided into five groups: control (C), FFA‐induced model (FFA), and FFA + IPA (15, 25, and 35 μM). Hepatic lipid accumulation was determined concurrently by Oil Red O staining and quantification of total cholesterol (TC) and triglyceride (TG) levels. Protein levels of lipogenesis markers and key components of the AHR and AMPK pathways were analyzed by Western blot.

To evaluate whether the effect of IPA in reducing lipid deposition depends on AMPK, cells were divided into six groups: C, FFA, FFA + IPA, AMPKint (AMPK inhibitor, AMPK‐IN‐3, 1 μM), FFA + AMPKint, and FFA + AMPKint + IPA. Lipid levels and AMPK pathway were examined.

To evaluate whether IPA activates the AMPK signaling pathway through AHR, cells were assigned to six groups: C, FFA, FFA + IPA (35 μM), AHRint (AHR inhibitor, AHR antagonist 5 free base, 1 μM), FFA + AHRint, and FFA + AHRint + IPA. Lipid content, AMPK pathway, and AHR nuclear translocation were evaluated.

To evaluate the effect of AMPK activation on the nuclear translocation of AHR, cells were divided into six groups: C, FFA, FFA + IPA, AKN (AMPK agonist, Ampkinone, 10 μM), FFA + AKN, and FFA + IPA + AHRint + AKN. Lipid accumulation and AHR nuclear translocation were assessed.

### 2.5 Transcriptome Analysis

Total RNA was isolated using Trizol reagent, with its integrity verified by agarose gel electrophoresis. Subsequently, transcriptome libraries were constructed for sequencing. Based on the results of the differential expression analysis, genes with a |log_2_FC| > 1 and FDR < 0.05 were identified as statistically significant DEGs. A volcano plot was generated to visualize the identified DEGs. GSEA was performed to delineate pathway enrichment. The input expression matrix was analyzed with the Signal2Noise metric for gene ranking. Gene set enrichment was analyzed using default parameters, with significance determined by applying predefined thresholds (|NES| > 1, nominal *p*‐value < 0.05, FDR *q*‐value < 0.25).

### 2.6 RNA Isolation and qPCR

Total RNA was isolated from tissues or cells using TRIzol reagent (Biosharp, China) according to the manufacturer's instructions, involving homogenization, chloroform phase separation, isopropanol precipitation, and ethanol washing. The resulting RNA was redissolved in RNase‐free water, and its purity (A260/A280 ≥ 1.8) was verified using a NanoDrop 2000 spectrophotometer (Thermo Fisher). Subsequent qPCR analysis was conducted with an SYBR Green PCR kit. The primer sequences used are listed in Tables 1 and 2.

**TABLE 1:** Primer sequences for genes in mouse.

Gene	Primer sequence	GenBank accession no.	Amplicon length (bp)
*Tnf‐α*	Forward CAGGCGGTGCCTATGTCTC Reverse CGATCACCCCGAAGTTCAGTAG	NM_013693.3	89
*Il‐6*	Forward TAGTCCTTCCTACCCCAATTTCC Reverse TTGGTCCTTAGCCACTCCTTC	NM_031168.2	76
*Il‐1β*	Forward TTCAGGCAGGCAGTATCACTC Reverse GAAGGTCCACGGGAAAGACAC	NM_008361.4	75
*SREBP‐1C*	Forward AACCTGCCCATCCACCGACTC Reverse TGCCTCCTCCACTGCCACA	NM_011480.4	322
*FAS*	Forward CTGTCAACCATGCCAACC Reverse CATCCACCCAAATCACCC	NM_007988.3	171
*GAPDH*	Forward TGTTTCCTCGTCCCGTAG Reverse CAATCTCCACTTTGCCACT	NM_007988.3	108
*β‐Actin*	Forward GGCTGTATTCCCCTCCATCG Reverse CCAGTTGGTAACAATGCCATGT	NM_007393.5	154

*Note:* Data are presented as the mean ± SD. The mRNA expression levels were determined by reverse transcription‐quantitative PCR. The expression of FAS and SREBP‐1c was normalized to GAPDH, while the expression of Tnf‐α, Il‐6, and Il‐1β was normalized to β‐actin. The 2^−ΔΔ*C*t^ method was used to calculate relative fold changes.

**TABLE 2:** Primer sequences for genes in HepG2 cells.

Gene	Primer sequence	GenBank accession no.	Amplicon length (bp)
*SREBP‐1C*	Forward ACAGTGACTTCCCTGGCCTAT Reverse GCATGGACGGGTACATCTTCAA	NM_004176.5	222
*FAS*	Forward CACAACTCCAAGGACACAGTCA Reverse CCCGGATCACCTTCTTGAGC	NM_004104.5	184
*β‐Actin*	Forward CCTGGCACCCAGCACAAT Reverse GGGCCGGACTCGTCATAC	NM_001101.5	144

*Note:* Data are presented as the mean ± SD. The mRNA expression levels of SREBP‐1C and FAS were determined by reverse transcription‐quantitative PCR and normalized to β‐actin expression. The 2^−ΔΔ*C*t^ method was used to calculate relative fold changes.

### 2.7 Protein Extraction and Western Blot Analysis

Total protein was extracted from mouse liver tissues using grinding beads in RIPA buffer, and from HepG2 cells by direct lysis in the same buffer. All samples were sonicated 3–5 times, then centrifuged (12,000 × g, 12 min, 4°C). Nuclear and cytoplasmic fractions were isolated using a commercial kit (Beyotime Biotechnology). After determination of protein concentration (BCA assay) and normalization, samples were denatured, electrophoresed on SDS‐PAGE gels (7.5%/10%), after transfer to PVDF membranes, blocking was performed with 5% skim milk. The membranes were then incubated sequentially with primary antibodies overnight at 4°C and HRP‐conjugated secondary antibodies for 1 h at room temperature. Primary antibody dilutions were as follows: β‐actin (1:5000), Lamin B1 (1:500), FAS (1:750), SREBPC (1:750), AHR (1:750, Affinity Biosciences), CYP1A1 (1:750), AMPK (1:500), and P‐AMPK (1:500). Primary antibodies against the target proteins (all from Affinity Biosciences except β‐actin, which was from Abcam) were used for immunoblotting. Goat Anti‐Rabbit IgG (HH) HRP (1:5000), Goat Anti‐Mouse IgG (H + L) HRP (1:5000, Affinity Biosciences). Bands were quantified with ImageJ, and analyzed with SPSS 25.0 software.

### 2.8 Statistical Analysis

All data are presented as mean ± standard deviation (SD). Each experimental group contained a minimum of three independent replicates. Statistical analyses were performed using SPSS 25.0 software. The normality of distribution and homogeneity of variances were assessed for all continuous data. When the data meet these two assumptions, the ANOVA method was used to compare the groups. Then, under the assumption of equal variance, the LSD test is carried out to compare multiple pairs. Otherwise, the nonparametric Kruskal–Wallis *H* test was utilized. Repeated measures ANOVA (RM‐ANOVA) was utilized for normally distributed continuous variables meeting sphericity assumptions. For every analysis, statistical significance was set at *p* < 0.05. All charts are drawn with the software GraphPad Prism 9.4.1 or BioGDP.

## 3 Results

### 3.1 IPA Attenuates FFA‐Induced Lipid Accumulation in HepG2 Cells

**FIGURE 1:**
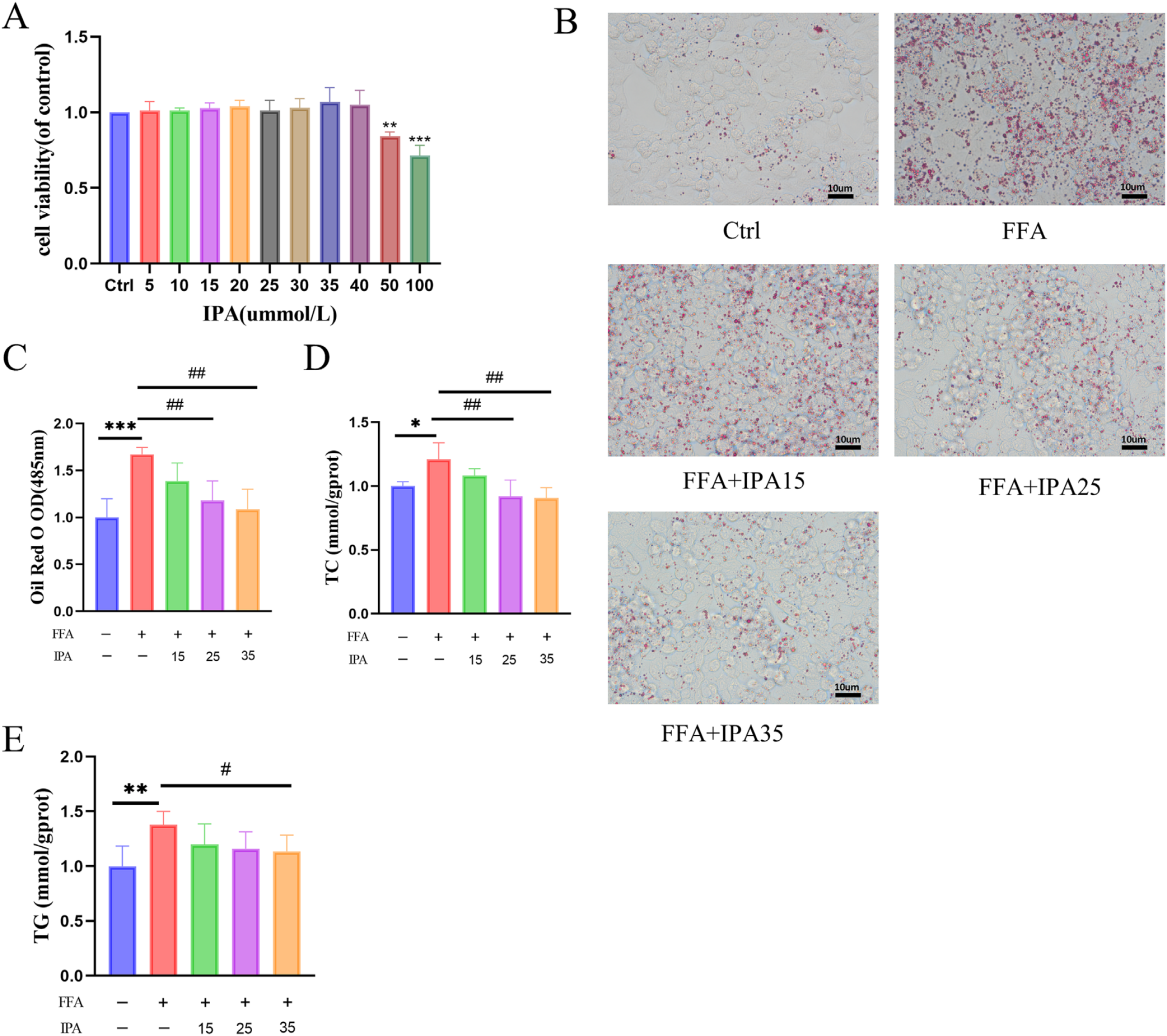
The influence of IPA on HepG2 cellular lipid deposition. (A) The effect of IPA treatment on the proliferation of HepG2 cells. (B, C) The effect of IPA treatment on lipid droplet accumulation in HepG2 cells, scale bar = 10 μm, original magnification: ×40. (D, E) The effects of IPA treatment on TC and TG content in HepG2 cells. Data were presented as mean ± SD. Asterisk indicates significant differences between Ctrl and FFA (*p < 0.05; **p < 0.01; ***p < 0.001). Pound indicates significant differences between FFA and IPA (#*p* < 0.05; ##*p* < 0.01). Data are presented as fold change relative to the control group, which was set to 1.

To evaluate the potential effects of different concentrations of IPA on HepG2 cytotoxicity, cell viability was assessed using the CCK‐8 assay. The results demonstrated that HepG2 cell proliferation was not significantly inhibited by treatment with 15 μM, 25 μM, and 35 μM IPA (Figure 1A). Therefore, these concentrations were used in subsequent experiments. A lipid accumulation model in HepG2 cells was established using free fatty acids to evaluate the effect of IPA on lipid deposition. Under the microscope, it was found that many small oil droplets dyed red by Oil Red O appeared in the cells exposed to free fatty acids. This result confirmed that an in vitro model of hepatic steatosis had been successfully established. Notably, the number of Oil Red O‐stained lipid droplets was dose‐dependently reduced by increasing concentrations of IPA (15–35 μM) (Figure 1B,C). FFA treatment induced a marked increase in TC and TG levels relative to the control. However, IPA intervention at different concentrations (15–35 μM) induced a marked, dose‐dependent reduction in both lipid components. Particularly, 35 μM IPA treatment resulted in the most substantial decrease in TC and TG levels (Figure 1D,E). These results suggest that IPA can alleviate lipid deposition in HepG2 cells.

### 3.2 IPA Alleviates Lipid Accumulation Through the Activation of AMPK Signaling Pathway

**FIGURE 2:**
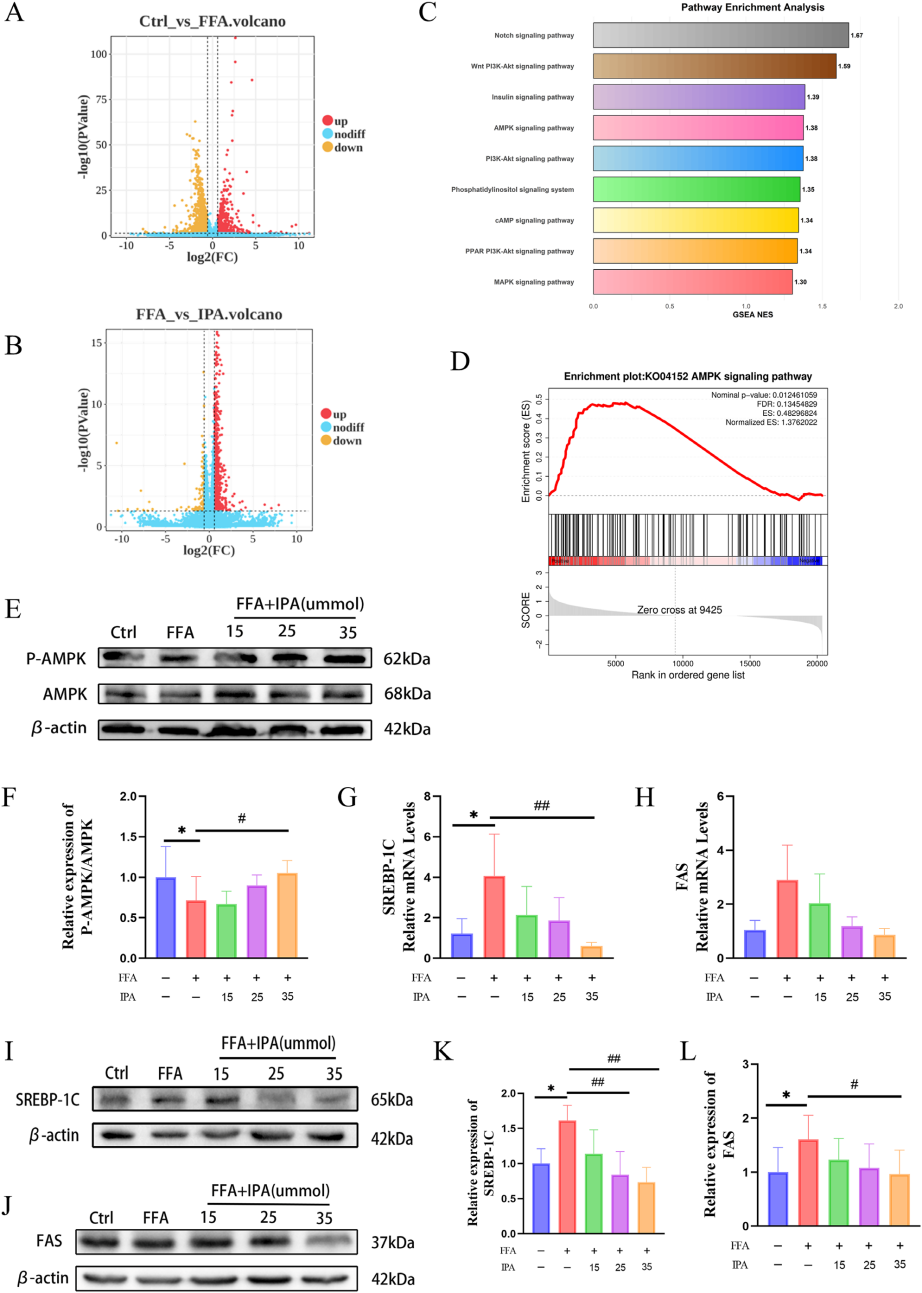
The effect of IPA treatment on the AMPK signaling pathway in HepG2 cells (A, B) Volcano plot. (C) The result of pathway enrichment derived from Gene Set Enrichment Analysis (GSEA). (D) The GSEA analysis results for the AMPK signaling pathway. (E, F) Western blotting analysis of the AMPK signaling pathway. (G, H) The mRNA expression of SREBP‐1C and FAS. (I–L) The protein expression of SREBP‐1C and FAS. Data were presented as mean ± SD. Asterisk indicated significant differences between Ctrl and FFA (*p < 0.05). Pound indicated significant differences between FFA and IPA (#*p* < 0.05; ##*p* < 0.01). Data are presented as fold change relative to the control group, which was set to 1.

To elucidate the mechanisms through which IPA alleviates lipid deposition, RNA sequencing was performed on HepG2 cells. The volcano plot revealed that a total of 2567 genes exhibited differential expression between the Control and FFA groups, among which 1789 genes were downregulated and 778 genes were upregulated. Furthermore, compared to the FFA group, IPA treatment resulted in 816 differentially expressed genes, including 91 downregulated and 725 upregulated genes (Figure 2A,B). According to the analysis of Gene Set Enrichment Analysis (GSEA), the AMPK signaling pathway was significantly enriched (Figure 2C,D). The complete GSEA gene lists and detailed pathway enrichment results are available in File S2 and File S3, respectively. As a well‐established central regulator of hepatic lipid metabolism and insulin sensitivity, dysregulation of AMPK is widely recognized as a key event in the pathogenesis and progression of MAFLD. We therefore focused on the AMPK pathway as a major target for subsequent mechanistic studies.

**FIGURE 3:**
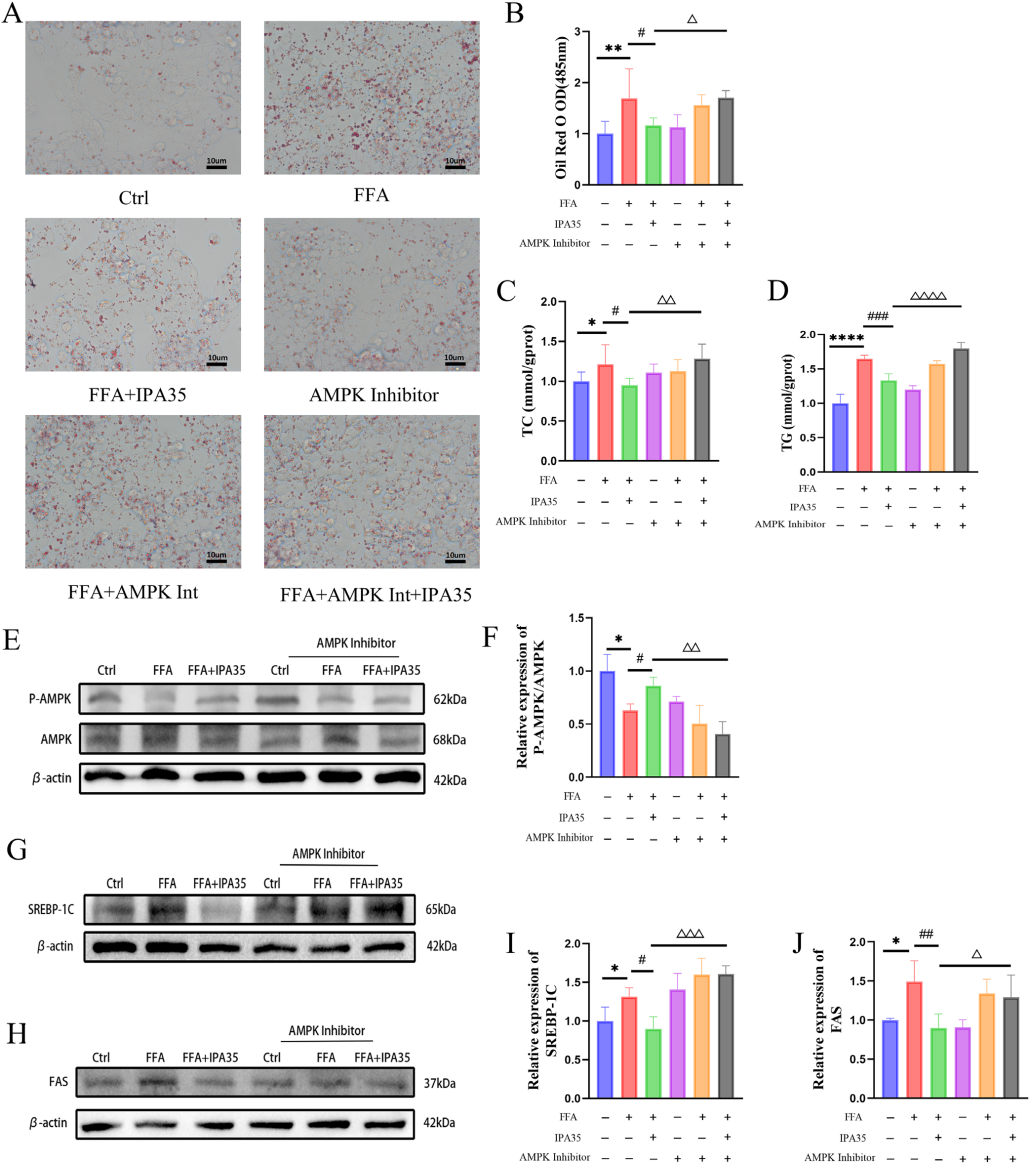
IPA ameliorates lipid accumulation by activating the AMPK signaling pathway. (A, B) The effect of AMPK inhibitor treatment on lipid droplet accumulation in HepG2 cells, scale bar = 10 μm, original magnification: ×40. (C, D) The effects of AMPK inhibitor treatment on *TC* and *TG* content in HepG2 cells. (E, F) Western blotting analysis of the AMPK signaling pathway. (G–J) The protein expression of *SREBP‐1C* and *FAS*. Data were presented as mean ± SD. Asterisk indicated significant differences between *Ctrl* and *FFA* (*p < 0.05; **p < 0.01; ****p < 0.0001). Pound indicated significant differences between FFA and IPA (#*p* < 0.05; ##*p* < 0.01;###*p* < 0.001). Triangles indicated significant differences between IPA and *AMPK Int + IPA* (△*p* < 0.05;△△*p* < 0.01; △△△*p* < 0.001; △△△△*p* < 0.0001). Data are presented as fold change relative to the control group, which was set to 1.

Western blot analysis revealed that FFA‐induced suppression of AMPK phosphorylation was markedly reversed upon IPA intervention, as evidenced by the restored P‐AMPK/AMPK ratio (Figure 2E,F). QPCR analysis demonstrated that free fatty acid (FFA) stimulation significantly upregulated sterol regulatory SREBP‐1c and FAS mRNA expression, while IPA treatment suppressed the expression of these lipogenic factors (Figure 2G,H). Consistent with mRNA findings, western blot analysis showed FFA markedly increased SREBP‐1C and FAS protein levels, which were dose‐dependently reduced by IPA (Figure 2I–L). To investigate the role of the AMPK pathway in IPA‐mediated suppression of steatosis, HepG2 cells were co‐treated with IPA and the AMPK inhibitor (AMPK‐IN‐3). Oil Red O staining showed that IPA greatly reduced the accumulation of lipid droplets, while the AMPK inhibitor abolished this protective effect (Figure 3A,B). Similarly, IPA decreased intracellular TC and TG levels, an effect that was reversed by the AMPK inhibitor (Figure 3C,D). Western blot analysis revealed that AMPK inhibitor treatment significantly reduced the ratios of P‐AMPK/AMPK (Figure 3E,F). Concurrently, the protein expression of key lipogenic genes, SREBP‐1c and FAS, was significantly upregulated (Figure 3G–J). Collectively, these results suggest that IPA alleviates lipid deposition by activating AMPK signaling.

### 3.3 IPA Activates AMPK Signaling Pathway Through AHR Nuclear Translocation

**FIGURE 4:**
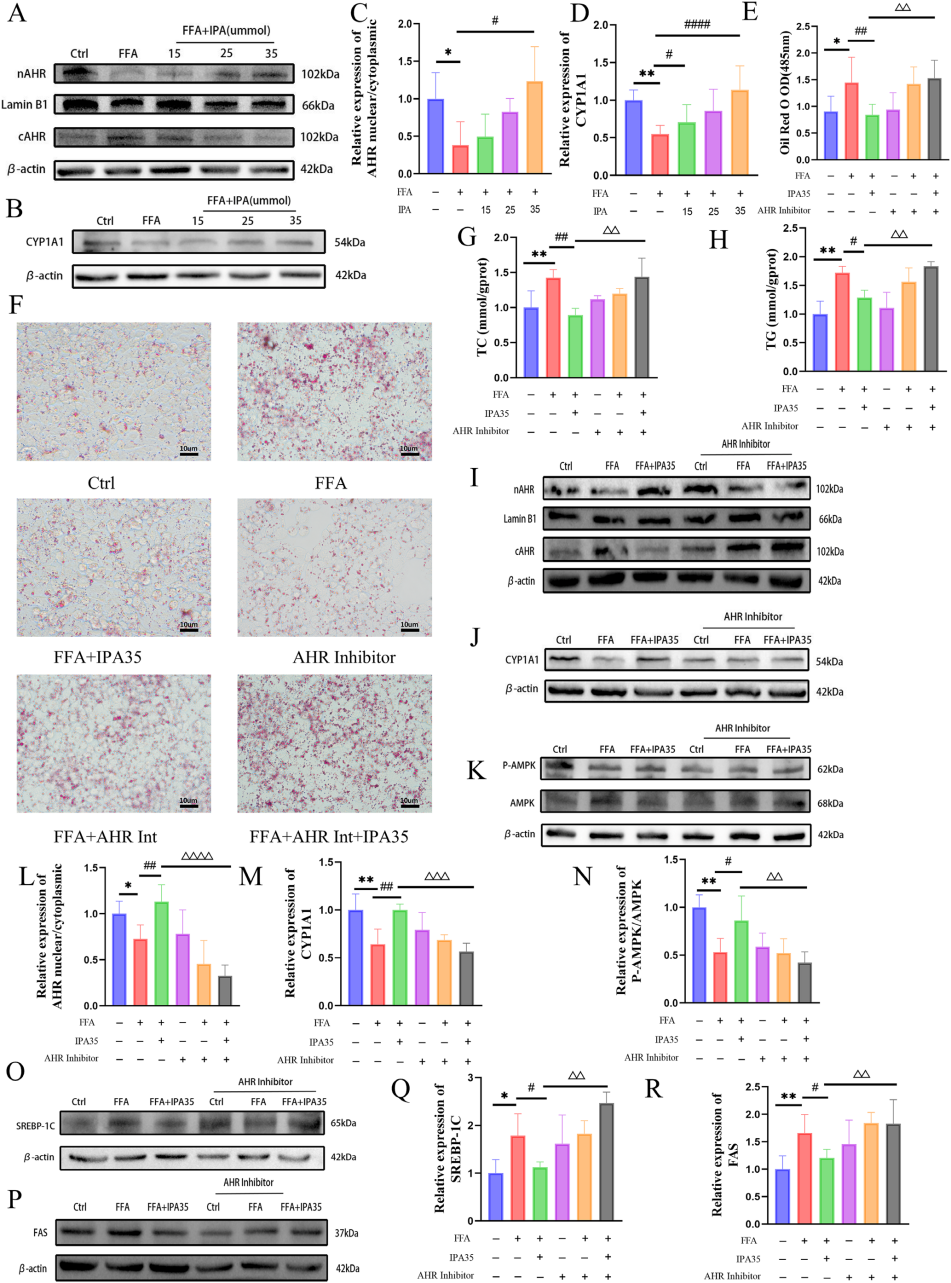
IPA activates AMPK signaling pathway in a manner dependent on AHR nuclear translocation. (A–D) Western blotting analysis of nuclear/cytoplasmic AHR and CYP1A1 protein levels. (E, F) The effect of AHR inhibitor treatment on lipid droplet accumulation in HepG2 cells, scale bar = 10 μm, original magnification: ×40. (G, H) The effects of AHR inhibitor treatment on TC and TG content in HepG2 cells. (I–N) Western blotting analysis of nuclear/cytoplasmic AHR and P‐AMPK/AMPK protein levels in AHR inhibitor‐treated HepG2 cells. (O–R) The protein expression of SREBP‐1C and FAS. Data were presented as mean ± SD. Asterisk indicated significant differences between control and FFA (*p < 0.05; **p < 0.01). Pound indicated significant differences between FFA and IPA (#*p* < 0.05; ##*p* < 0.01; ####*p* < 0.0001). Triangles indicated significant differences between IPA and AHR Int + IPA (△△*p* < 0.01; △△△*p* < 0.001;△△△△*p* < 0.0001). Data are presented as fold change relative to the control group, which was set to 1.

Given that IPA is a known activator of the AHR based on previous literature (Jalili et al. 2026), the impact of IPA on AHR nuclear transport and activation was examined. Western blot analysis revealed that FFA stimulation significantly suppressed AHR nuclear translocation and the protein expression of its downstream target gene CYP1A1. However, IPA intervention dose‐dependently promoted AHR nuclear translocation and upregulated CYP1A1 protein level (Figure 4A–D). To clarify the role of AHR in the improvement of lipid metabolism mediated by IPA, HepG2 cells were treated simultaneously with AHR inhibitor (AHR antagonist 5 free base) and IPA. Oil Red O staining demonstrated that IPA treatment markedly reduced lipid droplet accumulation, while AHR inhibition reversed this effect (Figure 4E,F). Cellular TC and TG levels were significantly decreased in IPA‐treated cells, but this improvement was substantially attenuated upon the addition of AHR inhibitor (Figure 4G,H). Western blot analysis demonstrated that treatment with the AHR inhibitor significantly reduced AHR nuclear translocation and the protein expression level of CYP1A1. It was worth noting that treatment with AHR inhibitor alone can inhibit the phosphorylation of AMPK, while the combination of IPA with AHR inhibitor will eliminate the activation effect of AMPK phosphorylation caused by IPA (Figure 4I–N). This co‐treatment also prominently upregulated the protein expression of SREBP‐1C and FAS (Figure 4O–R). These findings indicate that IPA‐mediated AMPK activation is contingent upon the nuclear translocation of AHR.

### 3.4 The Activation of AMPK Can Promote the Nuclear Translocation of AHR

**FIGURE 5:**
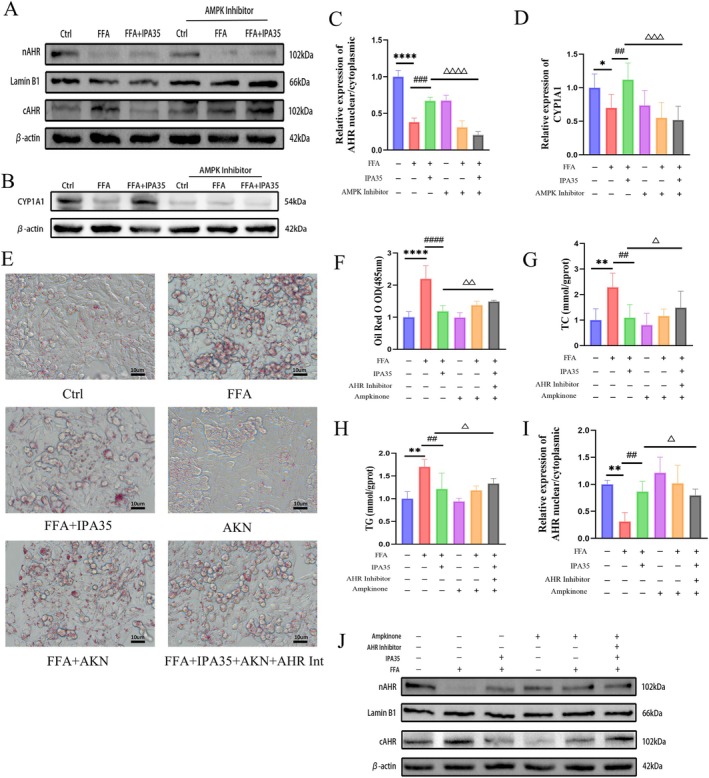
Phosphorylated AMPK promotes the nuclear translocation of AHR in HepG2 cells. (A–D) Western blotting analysis of nuclear/cytoplasmic AHR and CYP1A1 protein levels in AMPK inhibitor‐treated HepG2 cells. (E, F) The effect of AMPK agonist treatment on lipid droplet accumulation in HepG2 cells, scale bar = 10 μm, original magnification: ×40. (G, H) The effects of AMPK agonist treatment on TC and TG content in HepG2 cells. (I, J) Western blotting analysis of nuclear/cytoplasmic AHR levels in AMPK agonist‐treated HepG2 cells. Data were mean ± SD. Asterisk indicated significant differences between Ctrl and FFA (*p < 0.05; **p < 0.01; ****p < 0.0001). Pound indicated significant differences between FFA and IPA (#*p* < 0.05; ##*p* < 0.01; ###*p* < 0.001; ####*p* < 0.0001). Triangles indicated significant differences between IPA and AHR Int + IPA or FFA and AHR Int + IPA + AKN (△*p* < 0.05; △△*p* < 0.01; △△△*p* < 0.001; △△△△*p* < 0.0001). Data are presented as fold change relative to the control group, which was set to 1.

Previous results indicated that IPA‐induced activation of AMPK requires AHR nuclear translocation. However, treating HepG2 cells with the AMPK inhibitor alone could inhibit AHR nuclear translocation. Moreover, the combined use of IPA and the AMPK inhibitor could block IPA‐induced AHR activation. These findings suggest that AMPK phosphorylation might also influence AHR nuclear translocation. (Figure 5A–D). Therefore, AMPK agonist (Ampkinone) was used to further investigate the effects on AHR nuclear translocation and lipid deposition. Treatment with an AMPK agonist markedly reduced lipid accumulation in HepG2 cells, as evidenced by decreased Oil Red O staining and reduced TC/TG levels (Figure 5E–H). Notably, the combined use of IPA, AHR inhibitor, and AMPK agonist still significantly alleviated lipid deposition. Western blot analysis revealed that treating cells with the AMPK agonist alone could promote the nuclear translocation of AHR. Even in the presence of IPA and AHR inhibitor, the AMPK agonist was still able to maintain its effect on the nuclear translocation of AHR (Figure 5I–J). These results indicate that the activation of AHR can promote the phosphorylation of AMPK, and the activation of AMPK can further enhance the nuclear translocation and activity of AHR. There may be a potential bidirectional crosstalk interaction between AHR and AMPK. In HepG2 cells, IPA may enhance the improvement effect on lipid deposition through the potential bidirectional crosstalk between AHR and AMPK.

### 3.5 IPA Alleviates High‐Fat Diet‐Induced Hepatic Steatosis in Mice by Activating AHR and AMPK Signaling Pathway

**FIGURE 6:**
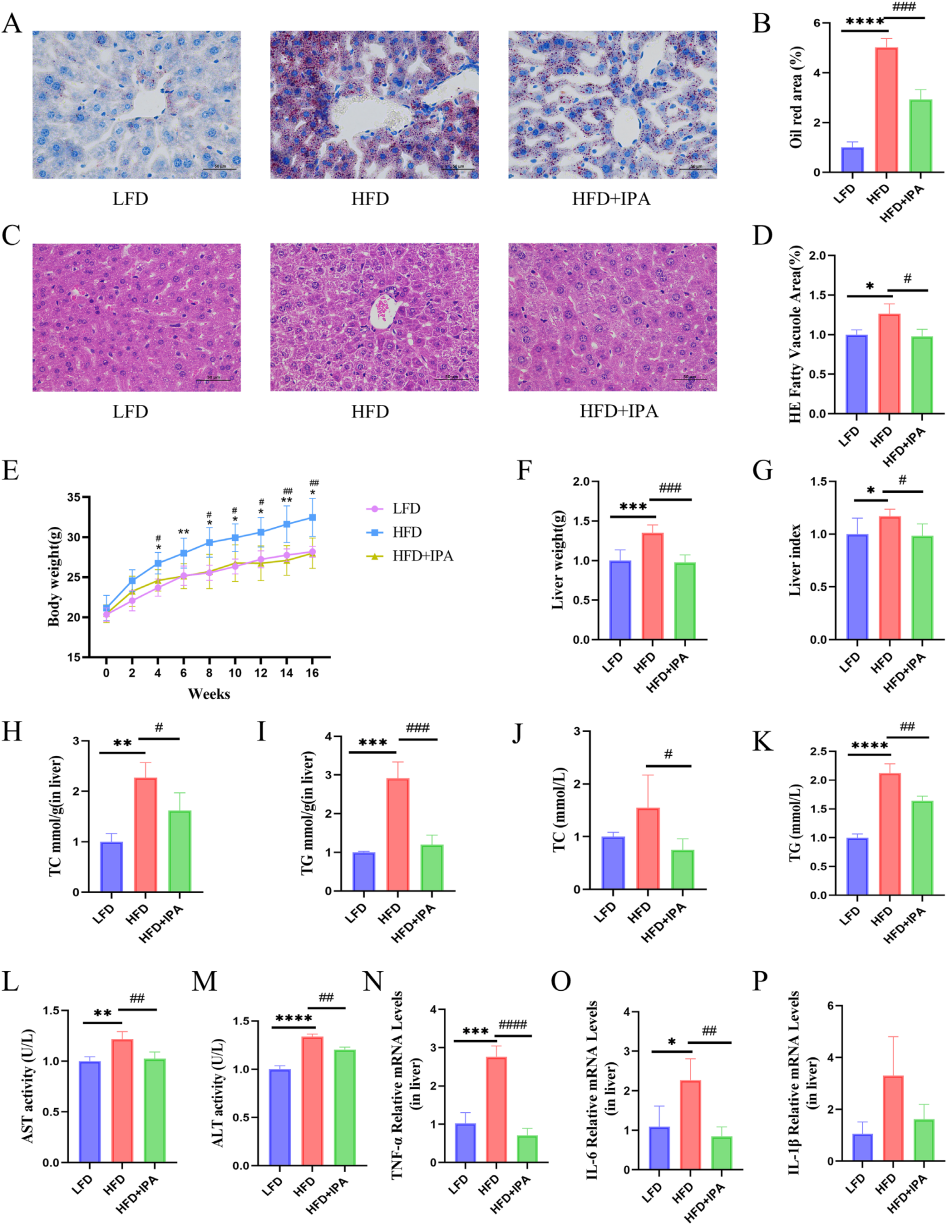
The effect of IPA on improving lipid deposition in mouse liver. (A, B) Oil Red O staining of lipid droplets in mouse liver tissue, scale bar = 50 μm, original magnification: ×40. (C, D) H&E staining and quantification of fat vacuole area in mouse liver tissue, scale bar = 50 μm, original magnification: ×40. (E–G) Body weight, liver weight, and liver index in mice. (H–K) Hepatic and serum TC and TG levels in mice. (L, M) Serum AST and ALT levels in mice. (N–P) The mRNA levels of TNF‐α, IL‐6 and IL‐1β in mouse liver. Data were presented as mean ± SD. Asterisk indicated significant differences between LFD and HFD (*p < 0.05; **p < 0.01; ***p < 0.001; ****p < 0.0001). Pound indicated significant differences between HFD and HFD + IPA (#*p* < 0.05; ##*p* < 0.01; ###*p* < 0.001; ####*p* < 0.0001). Data are presented as fold change relative to the control group, which was set to 1.

**FIGURE 7:**
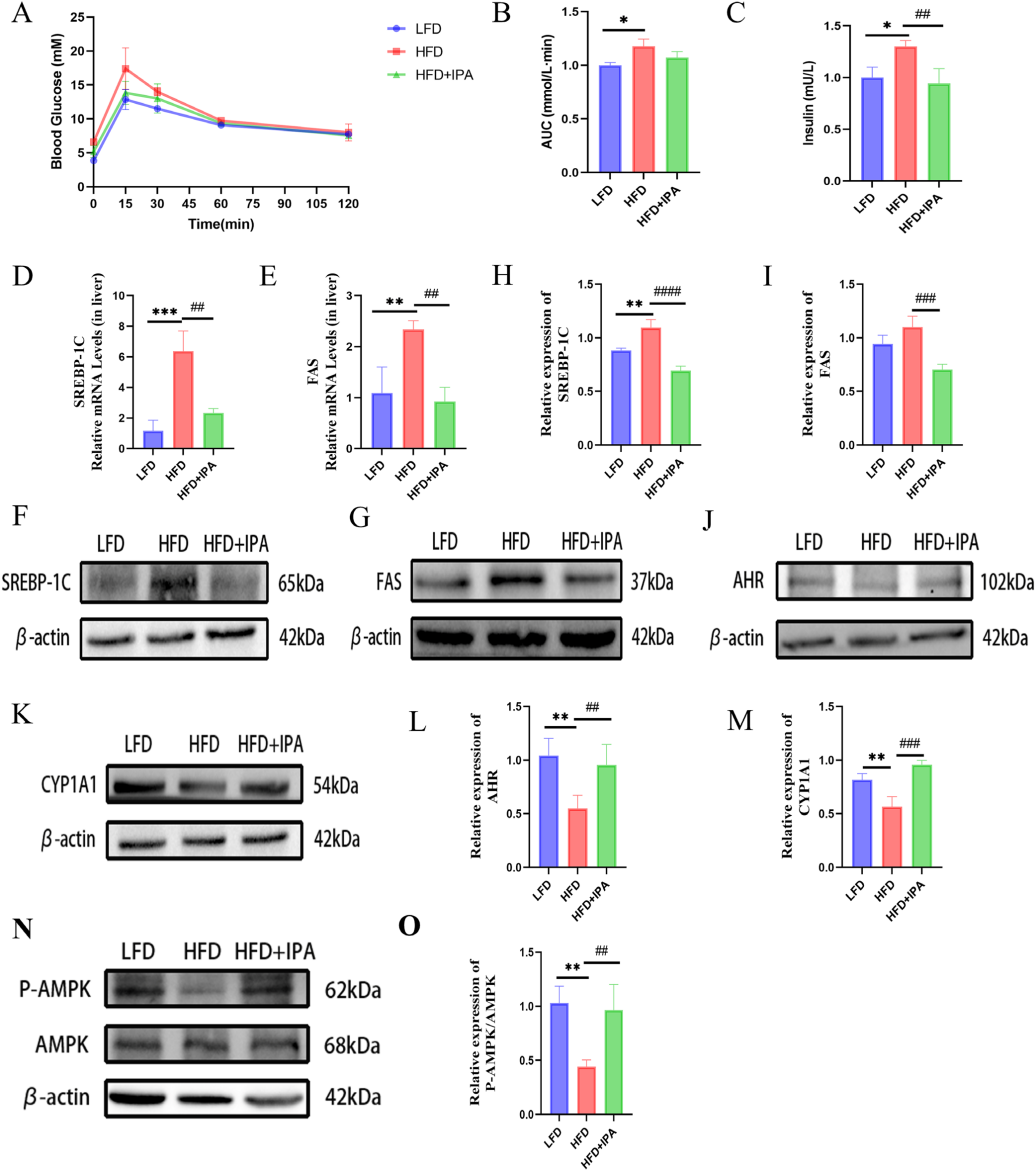
IPA ameliorates lipid accumulation through dual activation of both the AHR and AMPK signaling pathways. (A) Glucose tolerance test. (B) Corresponding AUC in mice fed ND, HFD, or HFD + IPA. (C) Fasting insulin levels. (D, E) The mRNA levels of SREBP‐1C and FAS in mouse liver. (F–J) Western blotting analysis of SREBP‐1C and FAS protein levels in mouse liver tissue. (K–N) Western blotting analysis of AHR and CYP1A1 protein levels in mouse liver tissue. (O, P) Western blotting analysis of P‐AMPK and AMPK protein levels in mouse liver tissue. Data were presented as mean ± SD. Asterisk indicated significant differences between LFD and HFD (*p < 0.05; **p < 0.01; ***p < 0.001). Pound indicated significant differences between HFD and HFD + IPA (#*p* < 0.05; ##*p* < 0.01; ###*p* < 0.001; ####*p* < 0.0001). Data are presented as fold change relative to the control group, which was set to 1.

Based on the evidence obtained from in vitro experiments, the effect of IPA on improving liver lipid deposition was further evaluated in the C57BL/6J mouse model. Oil Red O staining and histopathological analysis revealed that IPA supplementation markedly reduced hepatic lipid accumulation and ameliorated steatosis induced by a high‐fat diet (HFD) (Figure 6A–D). IPA also significantly reduced weight gain, liver weight gain, and liver index increase caused by high‐fat diet, while reducing the levels of total cholesterol and triglycerides in blood and liver (Figure 6E–K). In addition, IPA can alleviate liver injury, as reflected by reduced plasma AST and ALT activities (Figure 6L–M). It also reduced the expression of pro‐inflammatory cytokines (Tnf‐α, IL‐6, and IL‐1β) in the liver (Figure 6N–P). Furthermore, IPA treatment significantly reduced fasting insulin levels in HFD‐fed mice but did not significantly improve glucose tolerance (Figure 7A–C). Moreover, IPA downregulated mRNA and protein expression of lipogenic genes including Srebp‐1c and Fas (Figure 7D–I). Western blot analysis further showed that IPA increased protein levels of AHR and CYP1A1 in liver (Figure 7J–M). HFD‐induced suppression of AMPK phosphorylation was reversed by IPA treatment, resulting in increased p‐AMPK expression (Figure 7N–O). Collectively, these results indicate that IPA ameliorates HFD‐induced hepatic steatosis in mice by modulating AHR and AMPK signaling pathway.

## 4 Discussion

Metabolic dysfunction‐associated fatty liver disease (MAFLD) is a chronic liver condition closely linked to metabolic disorders and characterized by a complex pathogenesis involving multiple factors (He et al. 2025). Studies have shown that alterations in gut microbiota may influence metabolic function, thereby contributing to the development and progression of MAFLD (Yang et al. 2023). Recent studies have shown that tryptophan metabolites play a crucial role in the prevention and treatment of MAFLD. Some of these metabolites, such as indole‐3‐acetic acid (IAA), indole‐3‐propionic acid (IPA), melatonin, and serotonin, exhibit potential therapeutic effects (Chen and Ho 2023; Dongoran et al. 2023; Teunis et al. 2022). Specifically, IPA derived from gut microbiota via the indole pathway of tryptophan metabolism enhances intestinal barrier integrity by upregulating tight junction protein expression, thereby exerting anti‐inflammatory and antioxidant effects (Konopelski and Mogilnicka 2022). However, direct evidence that IPA can alleviate MAFLD by inhibiting lipid deposition remains limited. Therefore, this study aims to investigate the effects of IPA on lipogenesis and its associated metabolic signaling pathways.

In this study, lipid accumulation was induced in HepG2 cells through free fatty acid supplementation. Oil Red O staining demonstrated substantial lipid accumulation in hepatocytes, which correlated with markedly elevated hepatic TC and TG levels. Recent studies have indicated that dysregulation of TC and TG metabolism represents a pivotal pathogenic mechanism in MAFLD development (Fu et al. 2024). Our results revealed that IPA treatment reduced the lipid droplets and the levels of TC and TG, indicating that IPA can alleviate lipid deposition in HepG2 cells. The mouse model of hepatic steatosis induced by high‐fat diet was used to evaluate the improvement effect of IPA. The results showed that IPA reduced lipid accumulation in the liver as well as the levels of TC and TG. IPA administration suppressed hepatic protein levels of key lipogenic factors, Srebp‐1c and Fas. These findings indicated that IPA may improve MAFLD by directly influencing lipid synthesis.

The AMPK signaling pathway plays a crucial regulatory role in MAFLD (Li, Xu, et al. 2011; Palomer et al. 2025). AMPK activation significantly suppresses the expression of lipogenic genes, such as SREBP‐1c and FAS (Li et al. 2025), while upregulating fatty acid oxidation‐related genes, including CPT1 and PGC‐1α (Fang et al. 2022). Current literature lacks direct evidence demonstrating that IPA alleviates lipid accumulation in HepG2 cells via AMPK pathway activation. The analysis of RNA transcriptome showed that the AMPK signaling pathway was involved in the lipid metabolism regulated by IPA. The AMPK inhibitor was used to evaluate whether the effect of IPA on improving lipid deposition is dependent on the AMPK signaling pathway. The results showed that AMPK inhibitors can eliminate the improvement effect of IPA on lipid deposition, suggesting that the efficacy of IPA may be contingent upon AMPK signaling.

Given that IPA can serve as an endogenous ligand for AHR (Ghiboub et al. 2020), this study aimed to investigate the specific role of AHR in IPA‐mediated protective effects. AHR activation significantly modulates lipid metabolism, with the transcriptional upregulation of CYP1A1 serving as a canonical marker of AHR activation (Kim et al. 2022; Yin et al. 2016). AHR activation can ameliorate intracellular lipid accumulation in hepatocytes by regulating lipid synthesis‐related genes, such as SREBP‐1c and FASN (Tanos et al. 2012). In the FFA‐induced HepG2 cell model, we observed that reduced AHR nuclear translocation led to decreased expression of CYP1A1, confirming that FFA inhibits the AHR signaling pathway. In our results, IPA could activate AHR and promote the nuclear translocation. AHR inhibitor counteracted the effect of IPA in reducing lipid deposition, indicating that the lipid‐lowering effect of IPA is dependent on AHR activation.

Our results revealed that IPA can activate the AHR and AMPK signaling pathways. Therefore, we further investigated the roles of AHR and AMPK in the lipid‐lowering effect mediated by IPA. We found that IPA‐induced AMPK activation is dependent on AHR nuclear translocation, consistent with the recent studies highlighting the intricate crosstalk between AHR and AMPK signaling pathway (Patil et al. 2025). Our discovery suggests that AHR activation may play a regulatory role in the process of AMPK phosphorylation. However, the specific mechanism by which AHR regulates the phosphorylation of AMPK has not been further studied. Previous studies have established that LKB1 is the core upstream kinase of AMPK in the liver, activating AMPK by phosphorylating the Thr172 site, thereby regulating hepatic metabolic homeostasis (Qiu et al. 2023; Viollet et al. 2009). Recent studies have shown that AHR can regulate the transcription of LKB1, thereby influencing the fatty acid oxidation during the differentiation process of Treg cells (Zhang et al. 2023). Therefore, it is worth further investigating to determine whether there is a potential “AHR‐LKB1‐AMPK” regulatory axis that affects lipid deposition in the liver.

Previous studies have shown that AHR activation regulates energy metabolism pathways (Wang et al. 2024), consistent with our experimental findings that IPA‐induced AMPK activation requires AHR nuclear translocation. However, it has not been reported in the literature whether the phosphorylation of AMPK can in turn affect the nuclear translocation of AHR. Our results showed that AMPK activation further promotes AHR nuclear translocation, suggesting that there might be a positive feedback mechanism between AHR and AMPK. This mechanism is crucial for the improvement of lipid deposition effect mediated by IPA. The existence of a potential bidirectional crosstalk between AHR and AMPK holds great significance for understanding the complex regulatory network of cellular energy metabolism and environmental signal transduction. Therefore, a critical next step is to investigate how AMPK phosphorylation regulates AHR translocation. Furthermore, these results provide critical mechanistic insights and a theoretical framework for understanding how environmental factors interact with energy metabolism in obesity and other metabolic disorders. It may provide new targets and strategies for the treatment of metabolic diseases.

Beyond the AHR‐AMPK axis, IPA may exert additional hepatoprotective effects through complementary mechanisms. The anti‐inflammatory actions observed in our study (reduced hepatic IL‐6 and TNF‐α expression) suggest that IPA also modulates inflammatory signaling, likely via AHR‐mediated crosstalk with the NF‐κB pathway (Hu et al. 2025; Vogel et al. 2014). From a broader metabolic perspective, AMPK activation not only suppresses SREBP‐1c‐dependent lipogenesis but also enhances fatty acid oxidation and improves mitochondrial function, thereby alleviating hepatic steatosis and cellular stress (Lee et al. 2015; Zhang et al. 2024). Collectively, IPA modulates multiple complementary pathways to restore hepatic metabolic homeostasis. Through its lipid‐lowering, anti‐inflammatory, and mitochondrial‐protective effects, this gut microbial metabolite presents a promising spectrum of biological activities. These integrated and multifaceted effects highlight IPA as a promising multi‐target therapeutic candidate for MAFLD.

The concentrations of IPA used in vitro (15–35 μM) and the dosages administered in vivo (50 mg/kg/day) in this study were mainly based on their relevance to human physiology or clinical applications. First, the in vitro concentrations of IPA used in this study were designed to pharmacologically restore and moderately exceed basal serum levels observed in healthy humans (1–10 μM) (Zhang et al. 2022). Second, the in vivo dosage of IPA in mice, selected based on previous reports (Lu et al. 2025), translates to a human equivalent dose of approximately 4 mg/kg/day (≈250 mg/day for a 70 kg adult). This value falls well within the 50–500 mg/day range currently being evaluated in an ongoing Phase I/II clinical trial (NCT06674018), supporting the clinical feasibility and safety of IPA as a potential dietary supplement. Finally, the concentrations at which IPA exerted molecular and functional effects in vitro closely match the circulating levels achieved in vivo after oral administration (Jiang et al. 2022), indicating that our findings are biologically relevant and physiologically achievable.

This study has several limitations. First, all in vitro experiments were performed in HepG2 cells without verification in primary hepatocytes or organoids, and tissue‐specific knockout animals were not used; therefore, a direct causal relationship between IPA and its therapeutic benefits through the AHR/AMPK pathways cannot be firmly established. Second, the molecular crosstalk between AHR and AMPK remains undefined due to the lack of co‐immunoprecipitation, siRNA silencing, or phosphorylation‐site mapping. Third, the absence of gut microbiota and intestinal barrier analyses limits our understanding of the gut–liver axis, and species differences in AHR ligand sensitivity (Flaveny et al. 2010) caution against direct extrapolation, although clinical data have linked low circulating IPA to hepatic fibrosis (Sehgal et al. 2021). Fourth, this study focused on early‐stage MAFLD, leaving fibrotic NASH unexplored in vivo, despite anti‐fibrotic hints in human cohorts and in vitro HSC inactivation (Ilha et al. 2025; Sehgal et al. 2021). Fifth, we did not assess adipose tissue morphology, mitochondrial β‐oxidation, respiratory capacity, or lipophagy, and all animal experiments were conducted only in males. To address these gaps, future studies should validate our findings in primary hepatocytes or organoids, combine genetic (e.g., liver‐specific AHR/AMPK knockout) and pharmacological interventions to test causality, dissect the AHR–AMPK interaction using co‐immunoprecipitation, siRNA silencing, mutagenesis, and time‐course assays, integrate multi‐omics and fecal microbiota transplantation, employ humanized AHR mouse models and patient cohorts, evaluate IPA efficacy in fibrotic NASH and female animals, and measure mitochondrial function and lipid dynamics.

## 5 Conclusions

This study demonstrates that the gut microbiota‐derived metabolite IPA ameliorates hepatic lipid deposition by activating the AHR‐AMPK axis, thereby alleviating MAFLD. This discovery not only reveals a potential bidirectional crosstalk between AHR and AMPK, but also identifies the AHR/AMPK axis as a potential therapeutic target for MAFLD. In summary, this study offers new insights for the prevention and treatment of MAFLD.

## Author Contributions

**Yanting Huang:** writing – original draft, resources, investigation, data curation, methodology, validation, formal analysis. **Yalin Chen:** visualization, methodology, investigation. **Chujun Lin:** methodology, resources, validation, data curation. **Meimei Yu:** data curation, methodology, investigation. **Lijie Su:** writing – review and editing, visualization, supervision, project administration, funding acquisition, conceptualization. **Xin Chen:** visualization, formal analysis, data curation. **Jing Chen:** visualization, supervision, conceptualization, project administration, funding acquisition. **Hongliang Xue:** conceptualization, supervision, project administration, writing – review and editing, funding acquisition. **Shuhuan Li:** visualization, methodology, investigation. **Jiaxin Lu:** investigation, resources, validation, data curation.

## Funding

This study was funded by the National Natural Science Foundation of China (Grant No. 82304130), the Guangzhou Municipal Science and Technology Bureau Launch Program for Young Doctor [SL2024A04J01034], and the Guangzhou Medical University Scientific Research Capacity Improvement Project [2024SRP033, 2023SRP025].

## Ethics Statement

All animal experiments were conducted in accordance with the National Institutes of Health Guide for the Care and Use of Laboratory Animals and were approved by the Animal Experimental Ethics Committee of Guangzhou Medical University (Guangzhou, China; Approval No. GY2022‐084). All procedures were performed in compliance with relevant institutional and national guidelines and regulations for the care and use of laboratory animals.

## Conflicts of Interest

The authors declare no conflicts of interest.

## Supporting information


**Table S1:** Reagent: Main reagents and manufacturers.

**File S2.** Table S1. Detailed information of all reagents and antibodies used in this study, including suppliers.

**File S3.** Complete list of genes analyzed in the gene set enrichment analysis (GSEA).

## Data Availability

The data that support the findings of this study are available from the corresponding author upon reasonable request.

## References

[fsn372206-bib-0001] Alghamdi, W. , M. Mosli , and S. A. Alqahtani . 2024. “Gut Microbiota in MAFLD: Therapeutic and Diagnostic Implications.” Therapeutic Advances in Endocrinology and Metabolism 15: 20420188241242937. 10.1177/20420188241242937. 38628492 PMC11020731

[fsn372206-bib-0002] Badmus, O. O. , S. A. Hillhouse , C. D. Anderson , T. D. Hinds , and D. E. Stec . 2022. “Molecular Mechanisms of Metabolic Associated Fatty Liver Disease (MAFLD): Functional Analysis of Lipid Metabolism Pathways.” Clinical Science 136, no. 18: 1347–1366. 10.1042/CS20220572. 36148775 PMC9508552

[fsn372206-bib-0003] Bessone, F. , M. V. Razori , and M. G. Roma . 2019. “Molecular Pathways of Nonalcoholic Fatty Liver Disease Development and Progression.” Cellular and Molecular Life Sciences 76, no. 1: 99–128. 10.1007/s00018-018-2947-0. 30343320 PMC11105781

[fsn372206-bib-0004] Carotti, S. , K. Aquilano , F. Valentini , et al. 2020. “An Overview of Deregulated Lipid Metabolism in Nonalcoholic Fatty Liver Disease With Special Focus on Lysosomal Acid Lipase.” American Journal of Physiology. Gastrointestinal and Liver Physiology 319, no. 4: G469–G480. 10.1152/ajpgi.00049.2020. 32812776

[fsn372206-bib-0005] Chen, C.‐Y. , and H.‐C. Ho . 2023. “Roles of Gut Microbes in Metabolic‐Associated Fatty Liver Disease.” Tzu Chi Medical Journal 35, no. 4: 279–289. 10.4103/tcmj.tcmj_86_23. 38035063 PMC10683521

[fsn372206-bib-0006] Di Ciaula, A. , S. Passarella , H. Shanmugam , et al. 2021. “Nonalcoholic Fatty Liver Disease (NAFLD). Mitochondria as Players and Targets of Therapies?” International Journal of Molecular Sciences 22, no. 10: 5375. 10.3390/ijms22105375. 34065331 PMC8160908

[fsn372206-bib-0007] Dongoran, R. A. , F.‐C. Tu , and C.‐H. Liu . 2023. “Current Insights Into the Interplay Between Gut Microbiota‐Derived Metabolites and Metabolic‐Associated Fatty Liver Disease.” Tzu Chi Medical Journal 35, no. 4: 290–299. 10.4103/tcmj.tcmj_122_23. 38035056 PMC10683522

[fsn372206-bib-0008] Fang, C. , J. Pan , N. Qu , et al. 2022. “The AMPK Pathway in Fatty Liver Disease.” Frontiers in Physiology 13: 970292. 10.3389/fphys.2022.970292. 36203933 PMC9531345

[fsn372206-bib-0009] Feng, J. , L. MengHuan , Y. TingTing , Y. XueJie , and G. HaiNing . 2025. “Research Progress on AMPK in the Pathogenesis and Treatment of MASLD.” Frontiers in Immunology 16: 1558041. 10.3389/fimmu.2025.1558041. 40134423 PMC11932893

[fsn372206-bib-0010] Flaveny, C. A. , I. A. Murray , and G. H. Perdew . 2010. “Differential Gene Regulation by the Human and Mouse Aryl Hydrocarbon Receptor.” Toxicological Sciences 114, no. 2: 217–225. 10.1093/toxsci/kfp308. 20044593 PMC2840214

[fsn372206-bib-0011] Fu, Y. , Z. Wang , and H. Qin . 2024. “Examining the Pathogenesis of MAFLD and the Medicinal Properties of Natural Products From a Metabolic Perspective.” Metabolites 14, no. 4: 218. 10.3390/metabo14040218. 38668346 PMC11052500

[fsn372206-bib-0012] Geng, F. , and D. Guo . 2024. “SREBF1/SREBP‐1 Concurrently Regulates Lipid Synthesis and Lipophagy to Maintain Lipid Homeostasis and Tumor Growth.” Autophagy 20, no. 5: 1183–1185. 10.1080/15548627.2023.2275501. 37927089 PMC11135807

[fsn372206-bib-0013] Ghiboub, M. , C. M. Verburgt , B. Sovran , M. A. Benninga , W. J. de Jonge , and J. E. Van Limbergen . 2020. “Nutritional Therapy to Modulate Tryptophan Metabolism and Aryl Hydrocarbon‐Receptor Signaling Activation in Human Diseases.” Nutrients 12, no. 9: E2846. 10.3390/nu12092846. PMC7551725 32957545

[fsn372206-bib-0014] Gofton, C. , Y. Upendran , M.‐H. Zheng , and J. George . 2022. “MAFLD: How Is It Different From NAFLD?” Clinical and Molecular Hepatology 29: S17–S31. 10.3350/cmh.2022.0367. 36443926 PMC10029949

[fsn372206-bib-0015] Gourronc, F. A. , K. R. Markan , K. Kulhankova , et al. 2020. “Pdgfrα‐Cre Mediated Knockout of the Aryl Hydrocarbon Receptor Protects Mice From High‐Fat Diet Induced Obesity and Hepatic Steatosis.” PLoS One 15, no. 7: e0236741. 10.1371/journal.pone.0236741. 32730300 PMC7392206

[fsn372206-bib-0016] Han, Y. , Z. Hu , A. Cui , et al. 2019. “Post‐Translational Regulation of Lipogenesis via AMPK‐Dependent Phosphorylation of Insulin‐Induced Gene.” Nature Communications 10, no. 1: 623. 10.1038/s41467-019-08585-4. PMC6367348 30733434

[fsn372206-bib-0017] Harley, G. , M. Katerelos , K. Gleich , et al. 2022. “Blocking AMPK Signalling to Acetyl‐CoA Carboxylase Increases Cisplatin‐Induced Acute Kidney Injury and Suppresses the Benefit of Metformin.” Biomedicine & Pharmacotherapy 153: 113377. 10.1016/j.biopha.2022.113377. 36076520

[fsn372206-bib-0018] He, L. , X. She , L. Guo , et al. 2025. “Hepatic AKAP1 Deficiency Exacerbates Diet‐Induced MASLD by Enhancing GPAT1‐Mediated Lysophosphatidic Acid Synthesis.” Nature Communications 16, no. 1: 4286. 10.1038/s41467-025-58790-7. PMC12062205 40341440

[fsn372206-bib-0019] Hu, M. , Y. Xu , H. Zhou , and X. He . 2025. “Gut Microbial Metabolites of Amino Acids in Liver Diseases.” Gut Microbes 17, no. 1: 2586328. 10.1080/19490976.2025.2586328. 41277458 PMC12645865

[fsn372206-bib-0020] Huang, F.‐C. 2024. “Therapeutic Potential of Nutritional Aryl Hydrocarbon Receptor Ligands in Gut‐Related Inflammation and Diseases.” Biomedicine 12, no. 12: 2912. 10.3390/biomedicines12122912. PMC11673835 39767818

[fsn372206-bib-0021] Huang, W. , K. Rui , X. Wang , et al. 2023. “The Aryl Hydrocarbon Receptor in Immune Regulation and Autoimmune Pathogenesis.” Journal of Autoimmunity 138: 103049. 10.1016/j.jaut.2023.103049. 37229809

[fsn372206-bib-0022] Hyun, C.‐K. 2021. “Molecular and Pathophysiological Links Between Metabolic Disorders and Inflammatory Bowel Diseases.” International Journal of Molecular Sciences 22, no. 17: 9139. 10.3390/ijms22179139. 34502047 PMC8430512

[fsn372206-bib-0023] Ilha, M. , R. Sehgal , J. Matilainen , et al. 2025. “Indole‐3‐Propionic Acid Promotes Hepatic Stellate Cells Inactivation.” Journal of Translational Medicine 23, no. 1: 253. 10.1186/s12967-025-06266-z. 40025530 PMC11871697

[fsn372206-bib-0024] Jalili, C. , F. Hosseinkhani , D. Dayer , M. R. Tabandeh , A. Abbasi , and T. Zamir Nasta . 2026. “Indole‐3‐Propionic Acid Function Through PXR and AhR, Molecular Signaling Pathways, and Antitoxic Role in Underlying Diseases.” Journal of Steroid Biochemistry and Molecular Biology 255: 106877. 10.1016/j.jsbmb.2025.106877. 41093016

[fsn372206-bib-0025] Jiang, H. , C. Chen , and J. Gao . 2022. “Extensive Summary of the Important Roles of Indole Propionic Acid, a Gut Microbial Metabolite in Host Health and Disease.” Nutrients 15, no. 1: 151. 10.3390/nu15010151. 36615808 PMC9824871

[fsn372206-bib-0026] Kim, Y. S. , B. Ko , D. J. Kim , et al. 2022. “Induction of the Hepatic Aryl Hydrocarbon Receptor by Alcohol Dysregulates Autophagy and Phospholipid Metabolism via PPP2R2D.” Nature Communications 13, no. 1: 6080. 10.1038/s41467-022-33749-0. PMC9568535 36241614

[fsn372206-bib-0027] Konopelski, P. , and I. Mogilnicka . 2022. “Biological Effects of Indole‐3‐Propionic Acid, a Gut Microbiota‐Derived Metabolite, and Its Precursor Tryptophan in Mammals' Health and Disease.” International Journal of Molecular Sciences 23, no. 3: 1222. 10.3390/ijms23031222. 35163143 PMC8835432

[fsn372206-bib-0028] Krishnan, S. , Y. Ding , N. Saeidi , et al. 2019. “Gut Microbiota‐Derived Tryptophan Metabolites Modulate Inflammatory Response in Hepatocytes and Macrophages.” Cell Reports 28, no. 12: 3285. 10.1016/j.celrep.2019.08.080. 31533048 PMC6935324

[fsn372206-bib-0029] Lee, J.‐H. , J. Y. Jung , E. J. Jang , et al. 2015. “Combination of Honokiol and Magnolol Inhibits Hepatic Steatosis Through AMPK‐SREBP‐1 c Pathway.” Experimental Biology and Medicine 240, no. 4: 508–518. 10.1177/1535370214547123. 25125496 PMC4935380

[fsn372206-bib-0030] Li, N. , Q.‐W. Chen , X.‐L. Gong , et al. 2025. “Metformin and Berberine Synergistically Improve NAFLD via the AMPK‐SREBP1‐FASN Signaling Pathway.” Scientific Reports 15, no. 1: 29400. 10.1038/s41598-025-15495-7. 40790070 PMC12339966

[fsn372206-bib-0031] Li, Y. , S. Innocentin , D. R. Withers , et al. 2011. “Exogenous Stimuli Maintain Intraepithelial Lymphocytes via Aryl Hydrocarbon Receptor Activation.” Cell 147, no. 3: 629–640. 10.1016/j.cell.2011.09.025. 21999944

[fsn372206-bib-0032] Li, Y. , S. Xu , M. M. Mihaylova , et al. 2011. “AMPK Phosphorylates and Inhibits SREBP Activity to Attenuate Hepatic Steatosis and Atherosclerosis in Diet‐Induced Insulin‐Resistant Mice.” Cell Metabolism 13, no. 4: 376–388. 10.1016/j.cmet.2011.03.009. 21459323 PMC3086578

[fsn372206-bib-0033] Lin, Y.‐H. , H. Luck , S. Khan , et al. 2019. “Aryl Hydrocarbon Receptor Agonist Indigo Protects Against Obesity‐Related Insulin Resistance Through Modulation of Intestinal and Metabolic Tissue Immunity.” International Journal of Obesity (2005) 43, no. 12: 2407–2421. 10.1038/s41366-019-0340-1. 30944419 PMC6892742

[fsn372206-bib-0034] Lu, J. , Y. Huang , Y. Zhang , et al. 2025. “Quercetin Ameliorates Obesity and Inflammation via Microbial Metabolite Indole‐3‐Propionic Acid in High Fat Diet‐Induced Obese Mice.” Frontiers in Nutrition 12: 1574792. 10.3389/fnut.2025.1574792. 40308638 PMC12040668

[fsn372206-bib-0035] Luo, Y. , Y. Hao , C. Sun , et al. 2025. “Gut‐Derived Indole Propionic Acid Alleviates Liver Fibrosis by Targeting Profibrogenic Macrophages via the Gut–Liver Axis.” Cellular & Molecular Immunology 22: 1414–1426. 10.1038/s41423-025-01339-x. 40926017 PMC12575719

[fsn372206-bib-0036] Marano, G. , S. Rossi , G. Sfratta , et al. 2025. “Gut Microbiota: A New Challenge in Mood Disorder Research.” Life 15, no. 4: 593. 10.3390/life15040593. 40283148 PMC12028401

[fsn372206-bib-0037] Mulero‐Navarro, S. , and P. M. Fernandez‐Salguero . 2016. “New Trends in Aryl Hydrocarbon Receptor Biology.” Frontiers in Cell and Developmental Biology 4: 45. 10.3389/fcell.2016.00045. 27243009 PMC4863130

[fsn372206-bib-0038] Natividad, J. M. , A. Agus , J. Planchais , et al. 2018. “Impaired Aryl Hydrocarbon Receptor Ligand Production by the Gut Microbiota Is a Key Factor in Metabolic Syndrome.” Cell Metabolism 28, no. 5: 737–749.e4. 10.1016/j.cmet.2018.07.001. 30057068

[fsn372206-bib-0039] Palomer, X. , J.‐R. Wang , C. Escalona , S. Wu , W. Wahli , and M. Vázquez‐Carrera . 2025. “Targeting AMPK as a Potential Treatment for Hepatic Fibrosis in MASLD.” Trends in Pharmacological Sciences 46, no. 6: 551–566. 10.1016/j.tips.2025.03.008. 40300935

[fsn372206-bib-0040] Panda, S. K. , V. Peng , R. Sudan , et al. 2023. “Repression of the Aryl‐Hydrocarbon Receptor Prevents Oxidative Stress and Ferroptosis of Intestinal Intraepithelial Lymphocytes.” Immunity 56, no. 4: 797–812.e4. 10.1016/j.immuni.2023.01.023. 36801011 PMC10101911

[fsn372206-bib-0041] Patil, N. Y. , I. Rus , F. Ampadu , et al. 2025. “Cinnabarinic Acid Protects Against Metabolic Dysfunction‐Associated Steatohepatitis by Activating Aryl Hydrocarbon Receptor‐Dependent AMPK Signaling.” American Journal of Physiology. Gastrointestinal and Liver Physiology 328, no. 4: G433–G447. 10.1152/ajpgi.00337.2024. 40062565 PMC12258133

[fsn372206-bib-0042] Perepechaeva, M. L. , and A. Y. Grishanova . 2020. “The Role of Aryl Hydrocarbon Receptor (AhR) in Brain Tumors.” International Journal of Molecular Sciences 21, no. 8: 2863. 10.3390/ijms21082863. 32325928 PMC7215596

[fsn372206-bib-0043] Qiu, B. , A. Lawan , C. E. Xirouchaki , et al. 2023. “MKP1 Promotes Nonalcoholic Steatohepatitis by Suppressing AMPK Activity Through LKB1 Nuclear Retention.” Nature Communications 14, no. 1: 5405. 10.1038/s41467-023-41145-5. PMC10480499 37669951

[fsn372206-bib-0044] Rinella, M. E. , J. V. Lazarus , V. Ratziu , et al. 2023. “A Multisociety Delphi Consensus Statement on New Fatty Liver Disease Nomenclature.” Journal of Hepatology 79, no. 6: 1542–1556. 10.1016/j.jhep.2023.06.003. 37364790

[fsn372206-bib-0045] Sehgal, R. , M. Ilha , M. Vaittinen , et al. 2021. “Indole‐3‐Propionic Acid, a Gut‐Derived Tryptophan Metabolite, Associates With Hepatic Fibrosis.” Nutrients 13, no. 10: 3509. 10.3390/nu13103509. 34684510 PMC8538297

[fsn372206-bib-0046] Shi, D. , Y. Cui , H. Liang , Q. Wei , J. Huang , and J. Cao . 2025. “Gut Microbiota‐Derived Tryptophan Metabolite Indole‐3‐Carboxaldehyde Enhances Intestinal Barrier Function via AhR/AMPK Signaling Activation.” Animal Bioscience 39: e0225. 10.5713/ab.25.0225. PMC12754468 40665732

[fsn372206-bib-0047] Simon, T. G. , B. Roelstraete , H. Khalili , H. Hagström , and J. F. Ludvigsson . 2021. “Mortality in Biopsy‐Confirmed Nonalcoholic Fatty Liver Disease: Results From a Nationwide Cohort.” Gut 70, no. 7: 1375–1382. 10.1136/gutjnl-2020-322786. 33037056 PMC8185553

[fsn372206-bib-0048] Steinberg, G. R. , and D. G. Hardie . 2023. “New Insights Into Activation and Function of the AMPK.” Nature Reviews. Molecular Cell Biology 24, no. 4: 255–272. 10.1038/s41580-022-00547-x. 36316383

[fsn372206-bib-0049] Tanos, R. , I. A. Murray , P. B. Smith , A. Patterson , and G. H. Perdew . 2012. “Role of the Ah Receptor in Homeostatic Control of Fatty Acid Synthesis in the Liver.” Toxicological Sciences 129, no. 2: 372–379. 10.1093/toxsci/kfs204. 22696238 PMC3491957

[fsn372206-bib-0050] Teunis, C. , M. Nieuwdorp , and N. Hanssen . 2022. “Interactions Between Tryptophan Metabolism, the Gut Microbiome and the Immune System as Potential Drivers of Non‐Alcoholic Fatty Liver Disease (NAFLD) and Metabolic Diseases.” Metabolites 12, no. 6: 514. 10.3390/metabo12060514. 35736447 PMC9227929

[fsn372206-bib-0051] Ullah, H. , S. Arbab , C. Chang , et al. 2025. “Gut Microbiota Therapy in Gastrointestinal Diseases.” Frontiers in Cell and Developmental Biology 13: 1514636. 10.3389/fcell.2025.1514636. 40078367 PMC11897527

[fsn372206-bib-0052] Vernon, G. , A. Baranova , and Z. M. Younossi . 2011. “Systematic Review: The Epidemiology and Natural History of Non‐Alcoholic Fatty Liver Disease and Non‐Alcoholic Steatohepatitis in Adults.” Alimentary Pharmacology & Therapeutics 34, no. 3: 274–285. 10.1111/j.1365-2036.2011.04724.x. 21623852

[fsn372206-bib-0053] Viollet, B. , B. Guigas , J. Leclerc , et al. 2009. “AMP‐Activated Protein Kinase in the Regulation of Hepatic Energy Metabolism: From Physiology to Therapeutic Perspectives.” Acta Physiologica (Oxford, England) 196, no. 1: 81–98. 10.1111/j.1748-1716.2009.01970.x. 19245656 PMC2956117

[fsn372206-bib-0054] Vogel, C. F. A. , E. M. Khan , P. S. C. Leung , et al. 2014. “Cross‐Talk Between Aryl Hydrocarbon Receptor and the Inflammatory Response: A Role for Nuclear Factor‐κB.” Journal of Biological Chemistry 289, no. 3: 1866–1875. 10.1074/jbc.M113.505578. 24302727 PMC3894361

[fsn372206-bib-0055] Wang, Z. , Y. Zhang , Z. Liao , M. Huang , and X. Shui . 2024. “The Potential of Aryl Hydrocarbon Receptor as Receptors for Metabolic Changes in Tumors.” Frontiers in Oncology 14: 1328606. 10.3389/fonc.2024.1328606. 38434684 PMC10904539

[fsn372206-bib-0056] Xu, X. , S. Sun , L. Liang , et al. 2021. “Role of the Aryl Hydrocarbon Receptor and Gut Microbiota‐Derived Metabolites Indole‐3‐Acetic Acid in Sulforaphane Alleviates Hepatic Steatosis in Mice.” Frontiers in Nutrition 8: 756565. 10.3389/fnut.2021.756565. 34722615 PMC8548612

[fsn372206-bib-0057] Yang, C. , J. Xu , X. Xu , et al. 2023. “Characteristics of Gut Microbiota in Patients With Metabolic Associated Fatty Liver Disease.” Scientific Reports 13, no. 1: 9988. 10.1038/s41598-023-37163-4. 37340081 PMC10281992

[fsn372206-bib-0058] Yin, J. , B. Sheng , Y. Qiu , K. Yang , W. Xiao , and H. Yang . 2016. “Role of AhR in Positive Regulation of Cell Proliferation and Survival.” Cell Proliferation 49, no. 5: 554–560. 10.1111/cpr.12282. 27523394 PMC6496171

[fsn372206-bib-0059] Younossi, Z. , Q. M. Anstee , M. Marietti , et al. 2018. “Global Burden of NAFLD and NASH: Trends, Predictions, Risk Factors and Prevention.” Nature Reviews. Gastroenterology & Hepatology 15, no. 1: 11–20. 10.1038/nrgastro.2017.109. 28930295

[fsn372206-bib-0060] Zhang, B. , M. Jiang , J. Zhao , Y. Song , W. Du , and J. Shi . 2022. “The Mechanism Underlying the Influence of Indole‐3‐Propionic Acid: A Relevance to Metabolic Disorders.” Frontiers in Endocrinology 13: 841703. 10.3389/fendo.2022.841703. 35370963 PMC8972051

[fsn372206-bib-0061] Zhang, J. , Z. Liu , X. Yin , E. Wang , and J. Wang . 2024. “NSC48160 Targets AMPKα to Ameliorate Nonalcoholic Steatohepatitis by Inhibiting Lipogenesis and Mitochondrial Oxidative Stress.” iScience 27, no. 1: 108614. 10.1016/j.isci.2023.108614. 38155777 PMC10753068

[fsn372206-bib-0062] Zhang, Q. , Y. Zhu , C. Lv , et al. 2023. “AhR Activation Promotes Treg Cell Generation by Enhancing Lkb1‐Mediated Fatty Acid Oxidation via the Skp2/K63‐Ubiquitination Pathway.” Immunology 169, no. 4: 412–430. 10.1111/imm.13638. 36930164

[fsn372206-bib-0063] Zhao, Z.‐H. , F.‐Z. Xin , Y. Xue , et al. 2019. “Indole‐3‐Propionic Acid Inhibits Gut Dysbiosis and Endotoxin Leakage to Attenuate Steatohepatitis in Rats.” Experimental & Molecular Medicine 51, no. 9: 1–14. 10.1038/s12276-019-0304-5. PMC6802644 31506421

[fsn372206-bib-0064] Zheng, Y. , S. Wang , J. Wu , and Y. Wang . 2023. “Mitochondrial Metabolic Dysfunction and Non‐Alcoholic Fatty Liver Disease: New Insights From Pathogenic Mechanisms to Clinically Targeted Therapy.” Journal of Translational Medicine 21, no. 1: 510. 10.1186/s12967-023-04367-1. 37507803 PMC10375703

