# Supplementary material for: Indole‐3‐Propionic Acid Alleviates Metabolic Dysfunction‐Associated Fatty Liver Disease via AHR/AMPK Signaling Activation
Source: Food Sci Nutr. 2026 Aug 7;14(8):e72206. doi: 10.1002/fsn3.72206 (PMC13449033; doi:10.1002/fsn3.72206)
Supplement: Supplementary file 1 [file FSN3-14-e72206-s003.docx]

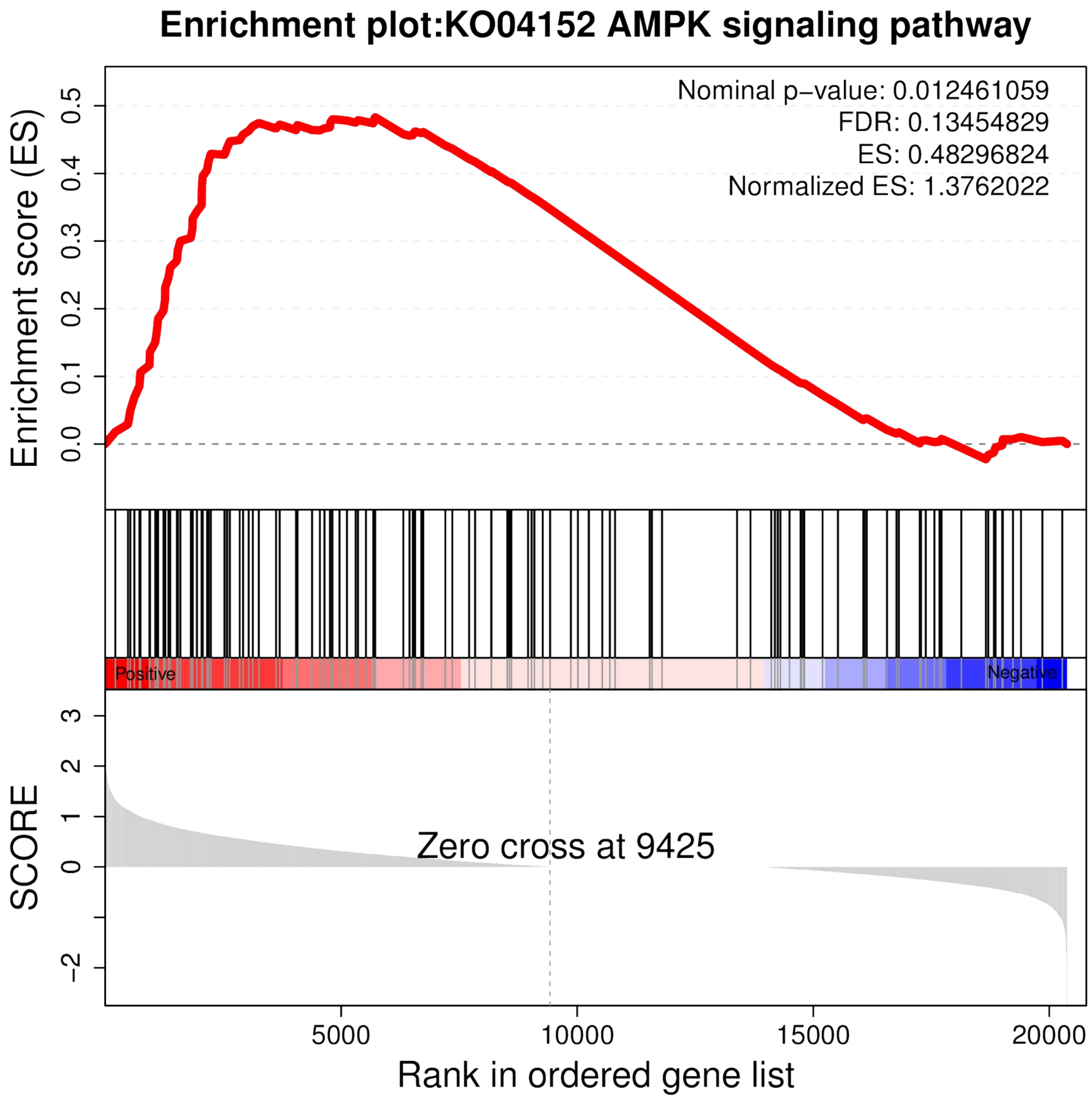
Enrichment plot:KO04152 AMPK signaling pathway
Nominal p−value: 0.012461059
FDR: 0.13454829
ES: 0.48296824
Normalized ES: 1.3762022
Enrichment score (ES)
0.0
0.1
0.2
0.3
0.4
0.5
Positive
Negative
SCORE
Zero cross at 9425
3
2
1
0
−2
5000
10000
15000
20000
Rank in ordered gene list

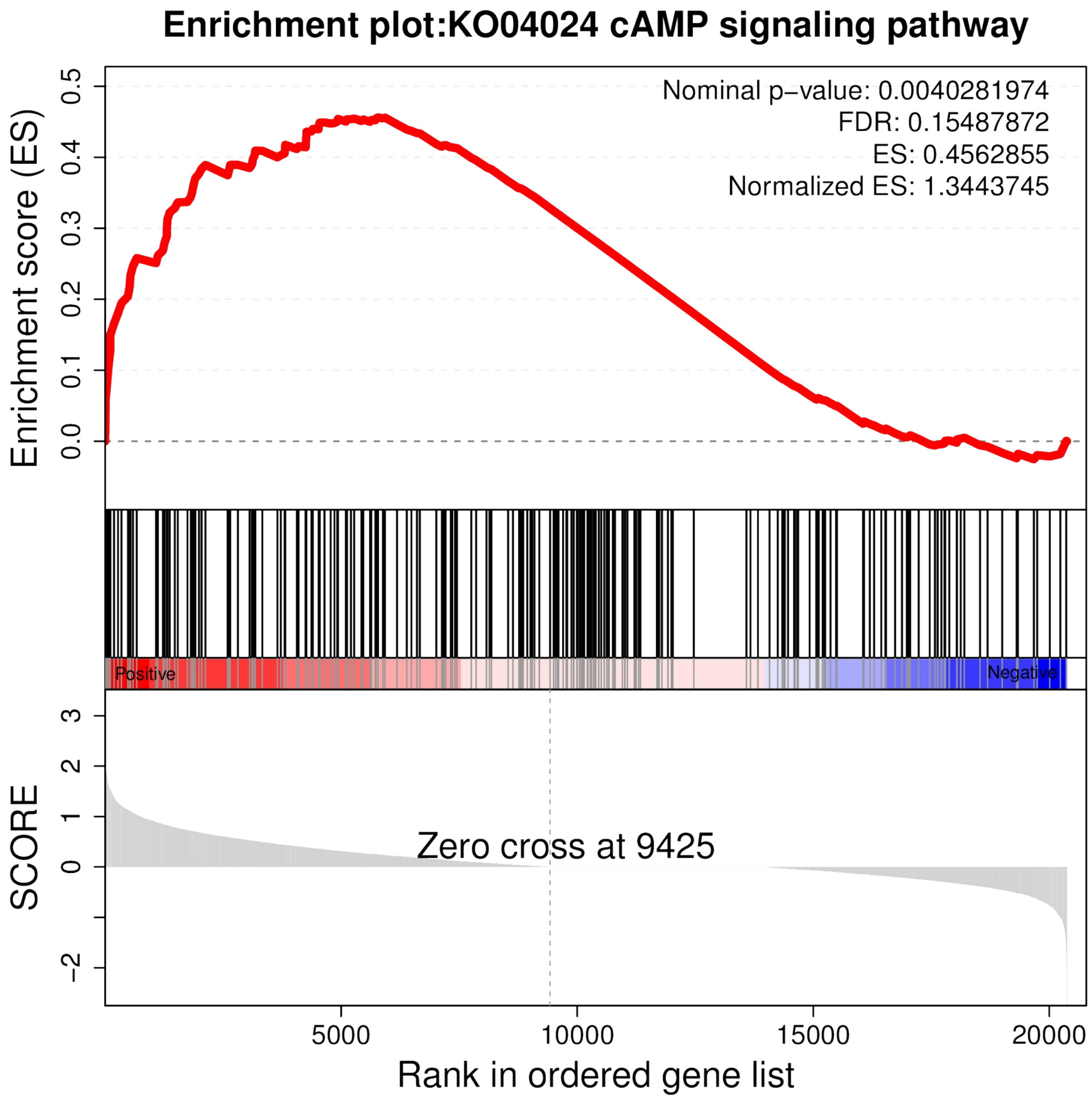
Enrichment plot:KO04024 cAMP signaling pathway
Nominal p−value: 0.0040281974
FDR: 0.15487872
ES: 0.4562855
Normalized ES: 1.3443745
Enrichment score (ES)
0.0
0.1
0.2
0.3
0.4
0.5
Positive
Negative
SCORE
Zero cross at 9425
3
2
1
0
−2
5000
10000
15000
20000
Rank in ordered gene list

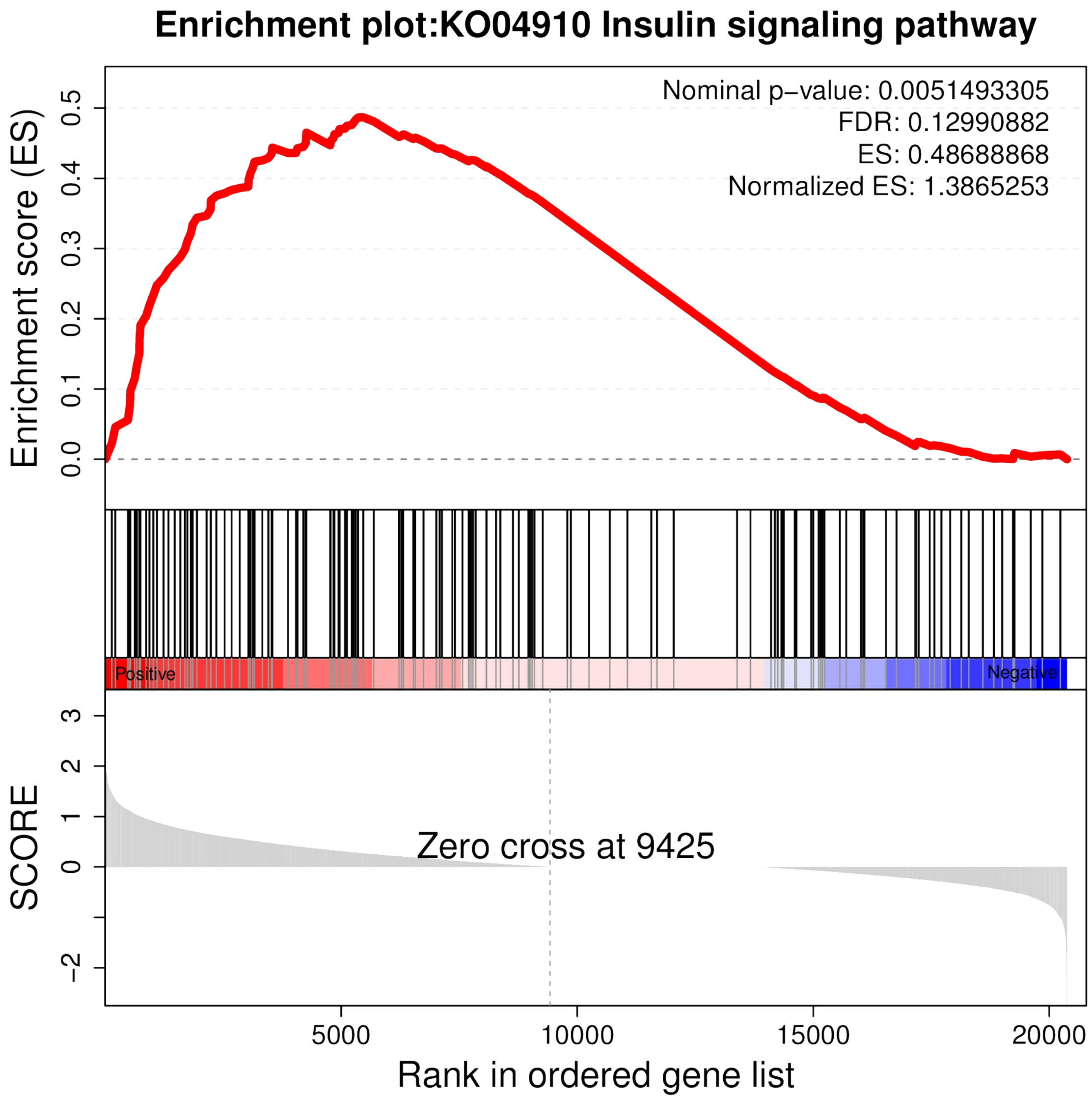

Enrichment plot:KO04910 Insulin signaling pathway
Nominal p−value: 0.0051493305
FDR: 0.12990882
ES: 0.48688868
Normalized ES: 1.3865253
Enrichment score (ES)
0.0
0.1
0.2
0.3
0.4
0.5
Positive
Negative
SCORE
Zero cross at 9425
3
2
1
0
−2
5000
10000
15000
20000
Rank in ordered gene list

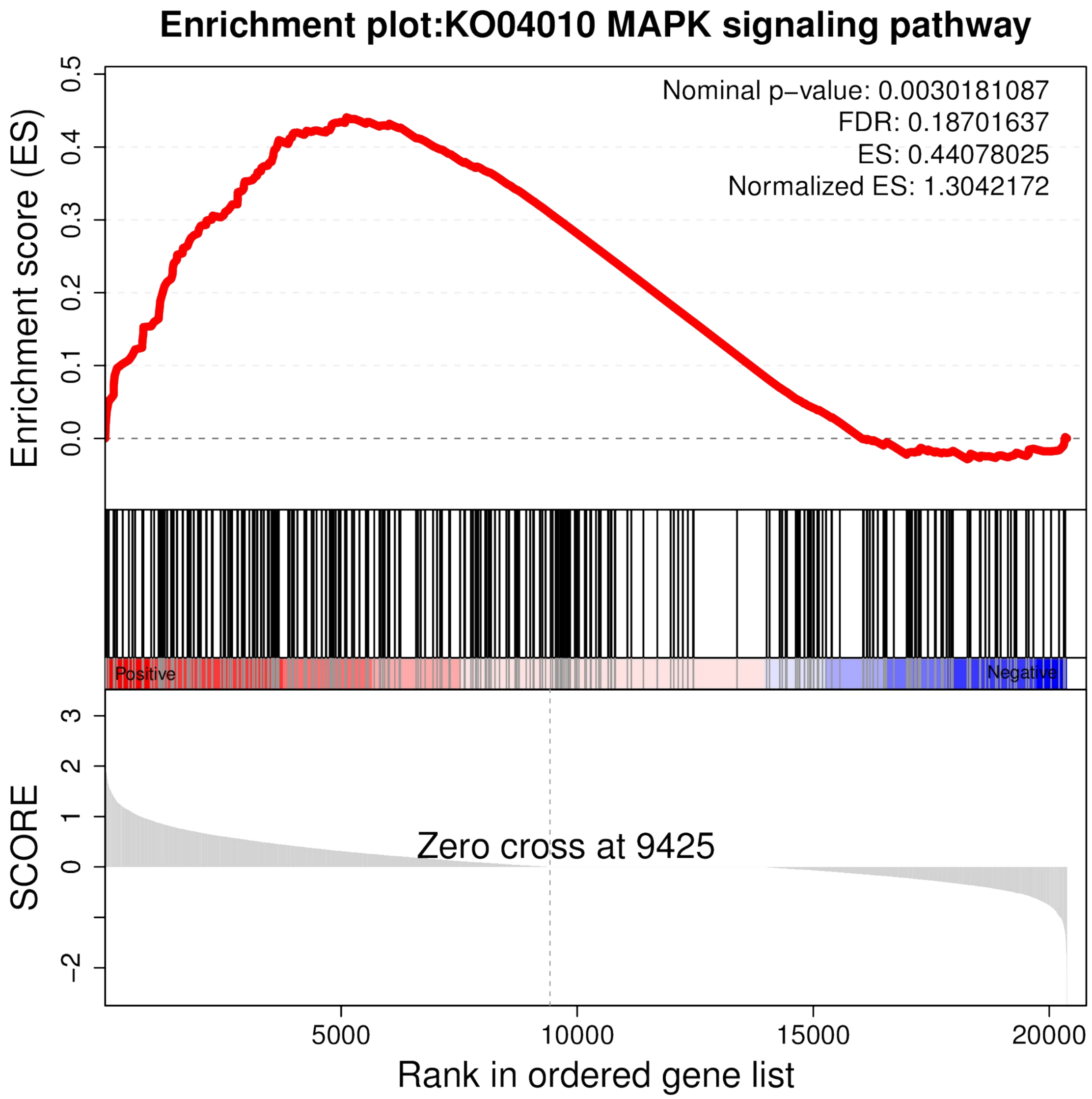
Enrichment plot:KO04010 MAPK signaling pathway
Nominal p−value: 0.0030181087
FDR: 0.18701637
ES: 0.44078025
Normalized ES: 1.3042172
Enrichment score (ES)
0.0
0.1
0.2
0.3
0.4
0.5
Positive
Negative
SCORE
Zero cross at 9425
−2
0
1
2
3
5000
10000
15000
20000
Rank in ordered gene list

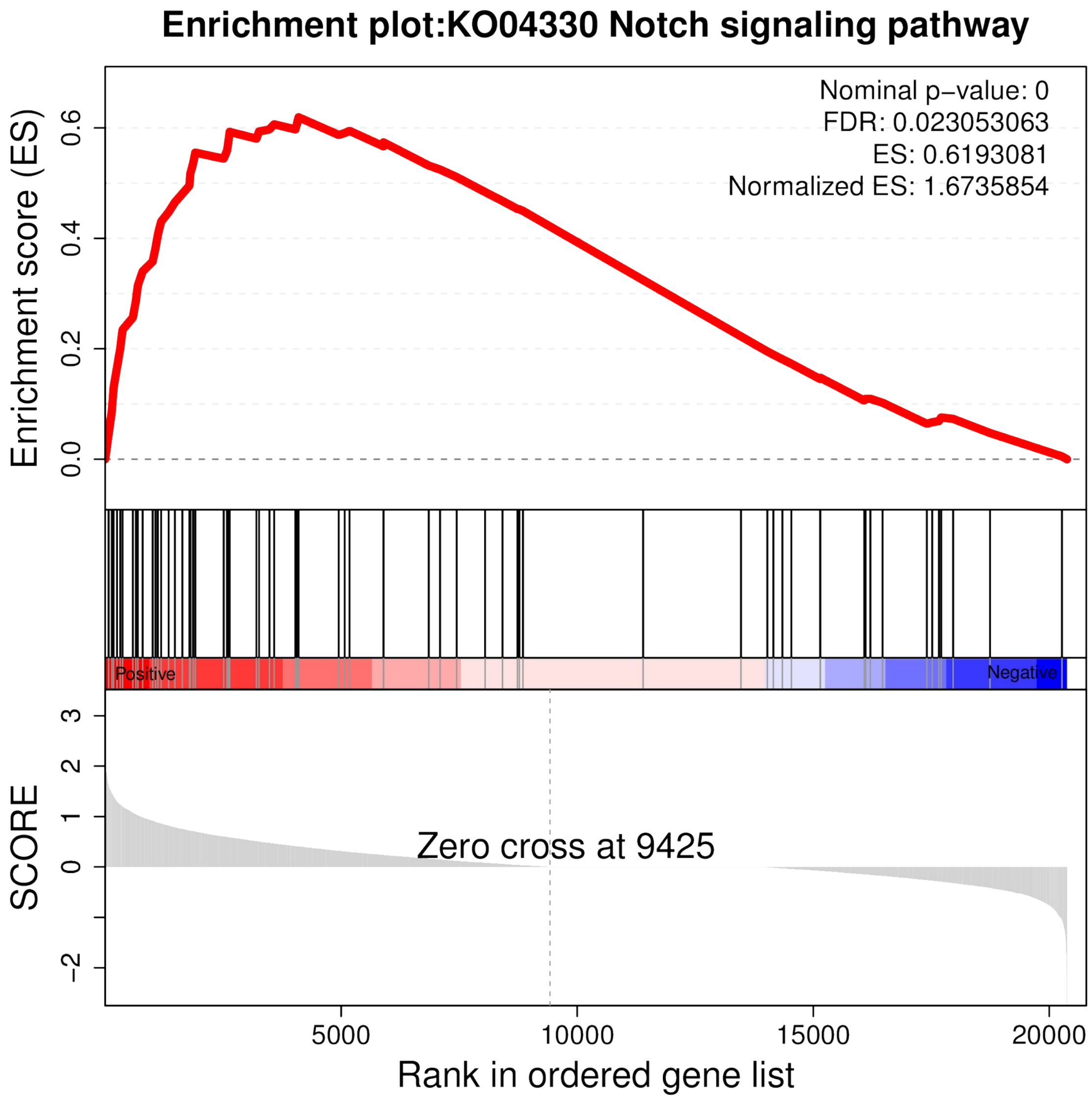

Enrichment plot:KO04330 Notch signaling pathway
Nominal p−value: 0
FDR: 0.023053063
ES: 0.6193081
Normalized ES: 1.6735854
Enrichment score (ES)
0.0
0.2
0.4
0.6
Positive
Negative
SCORE
3
2
1
0
−2
Zero cross at 9425
5000
10000
15000
20000
Rank in ordered gene list

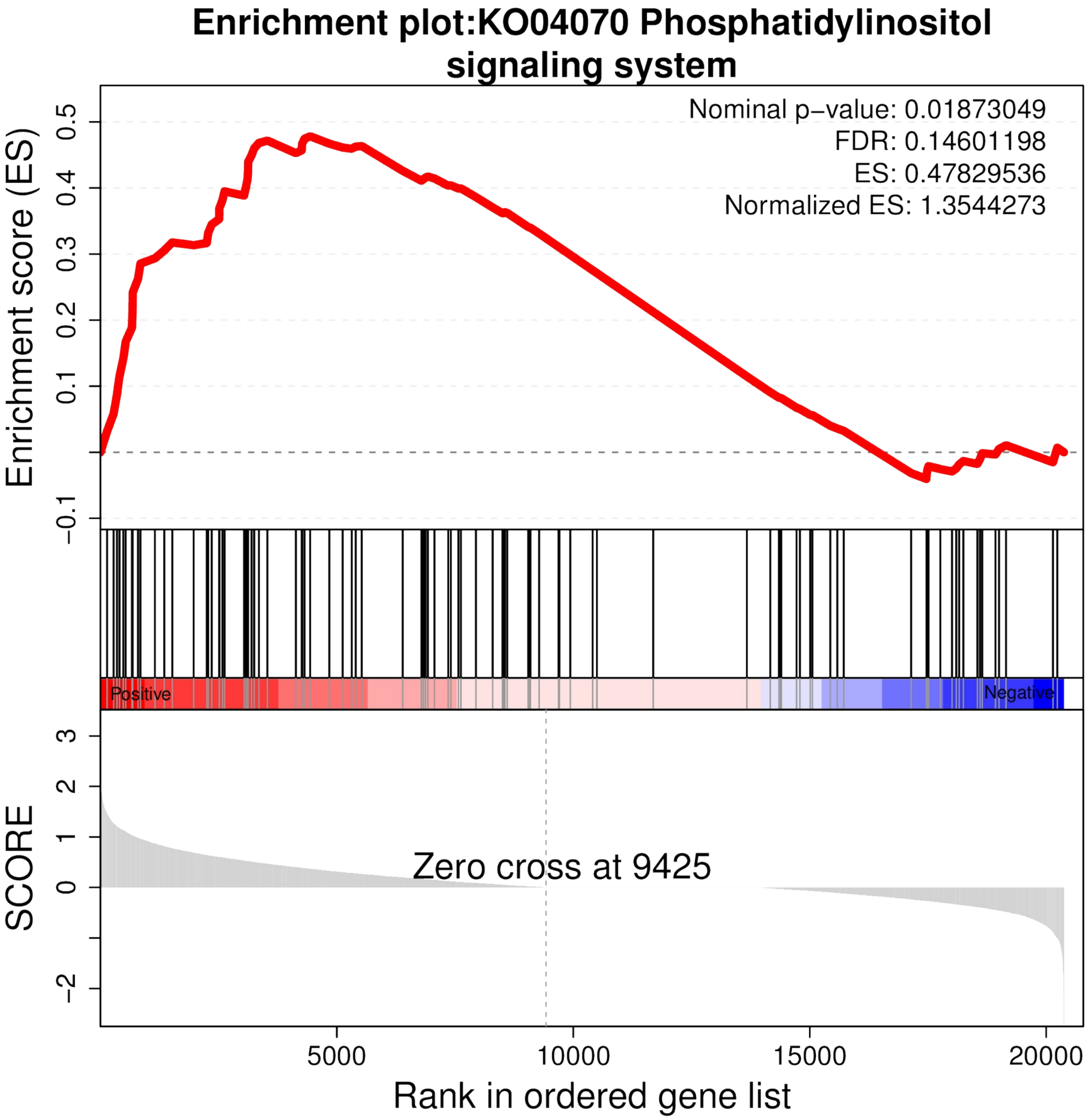

Enrichment plot:KO04070 Phosphatidylinositol signaling system
Nominal p−value: 0.01873049
FDR: 0.14601198
ES: 0.47829536
Normalized ES: 1.3544273
Enrichment score (ES)
0.5
0.4
0.3
0.2
0.1
−0.1
Positive
Negative
SCORE
3
2
1
0
−2
Zero cross at 9425
5000
10000
15000
20000
Rank in ordered gene list

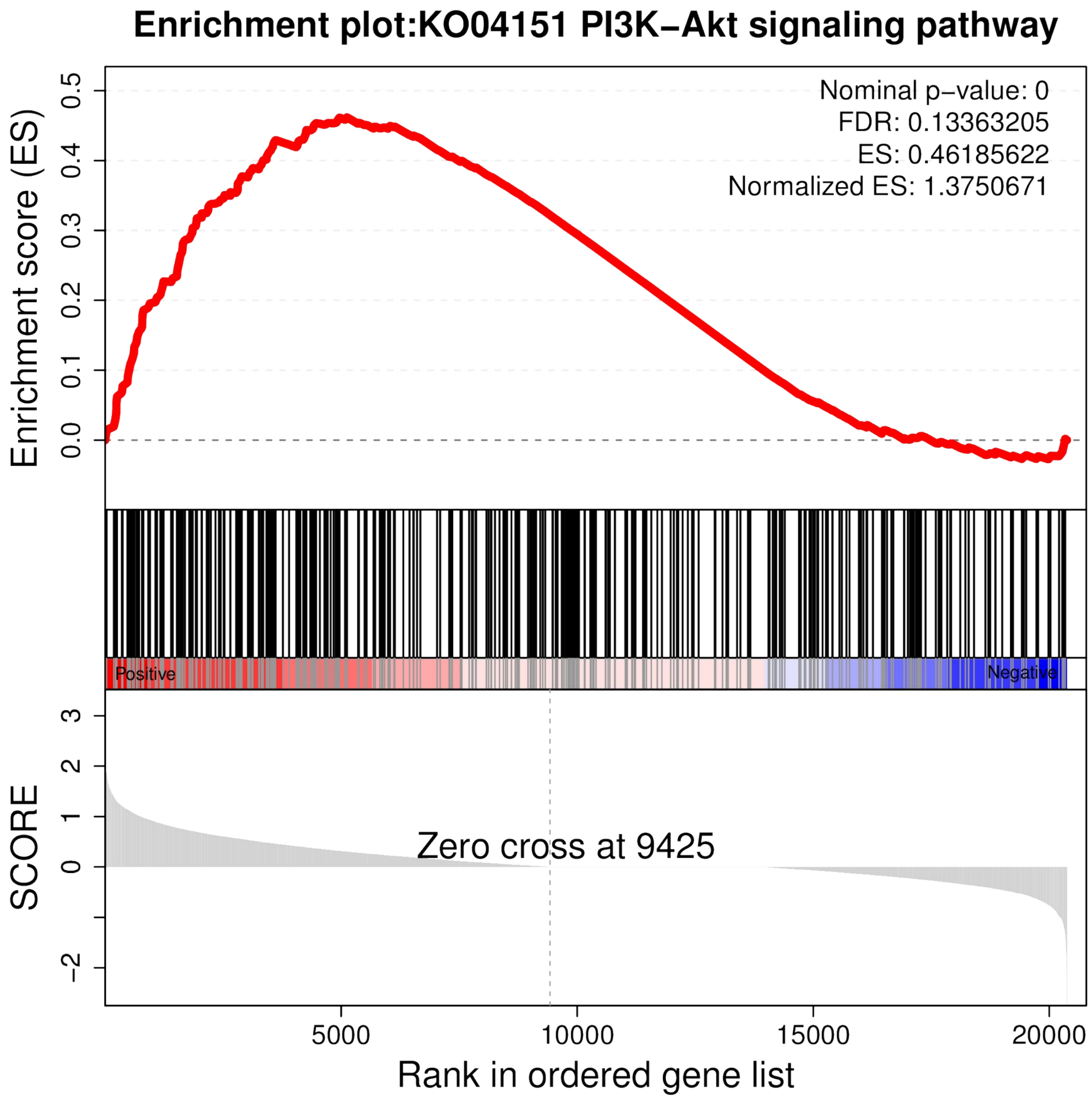
Enrichment plot:KO04151 PI3K−Akt signaling pathway
Nominal p−value: 0
FDR: 0.13363205
ES: 0.46185622
Normalized ES: 1.3750671
Enrichment score (ES)
0.0
0.1
0.2
0.3
0.4
0.5
Positive
Negative
SCORE
3
2
1
0
−2
Zero cross at 9425
5000
10000
15000
20000
Rank in ordered gene list

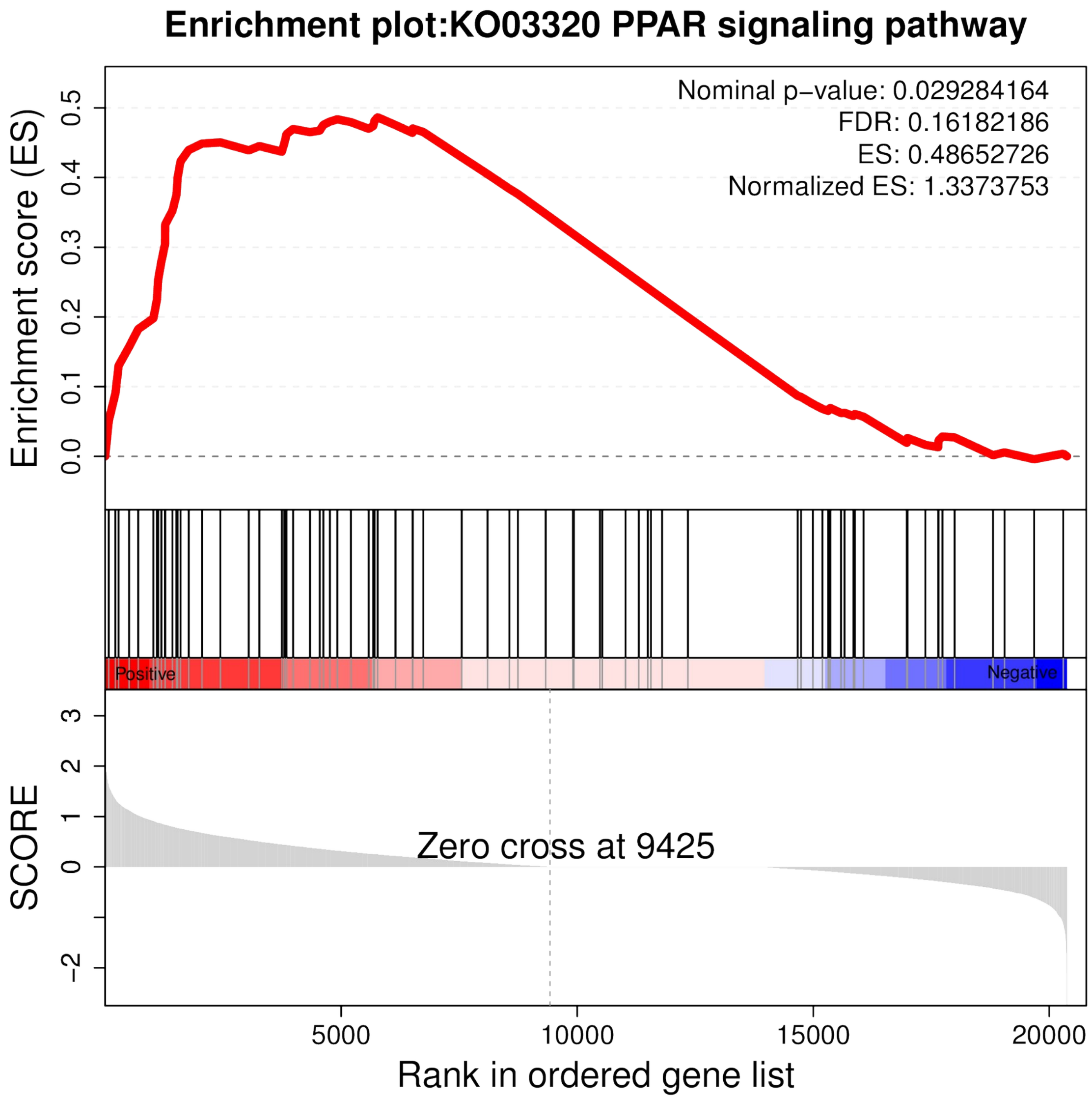
Enrichment plot:KO03320 PPAR signaling pathway
Nominal p−value: 0.029284164
FDR: 0.16182186
ES: 0.48652726
Normalized ES: 1.3373753
Enrichment score (ES)
0.0
0.1
0.2
0.3
0.4
0.5
Positive
Negative
SCORE
Zero cross at 9425
3
2
1
0
−2
5000
10000
15000
20000
Rank in ordered gene list

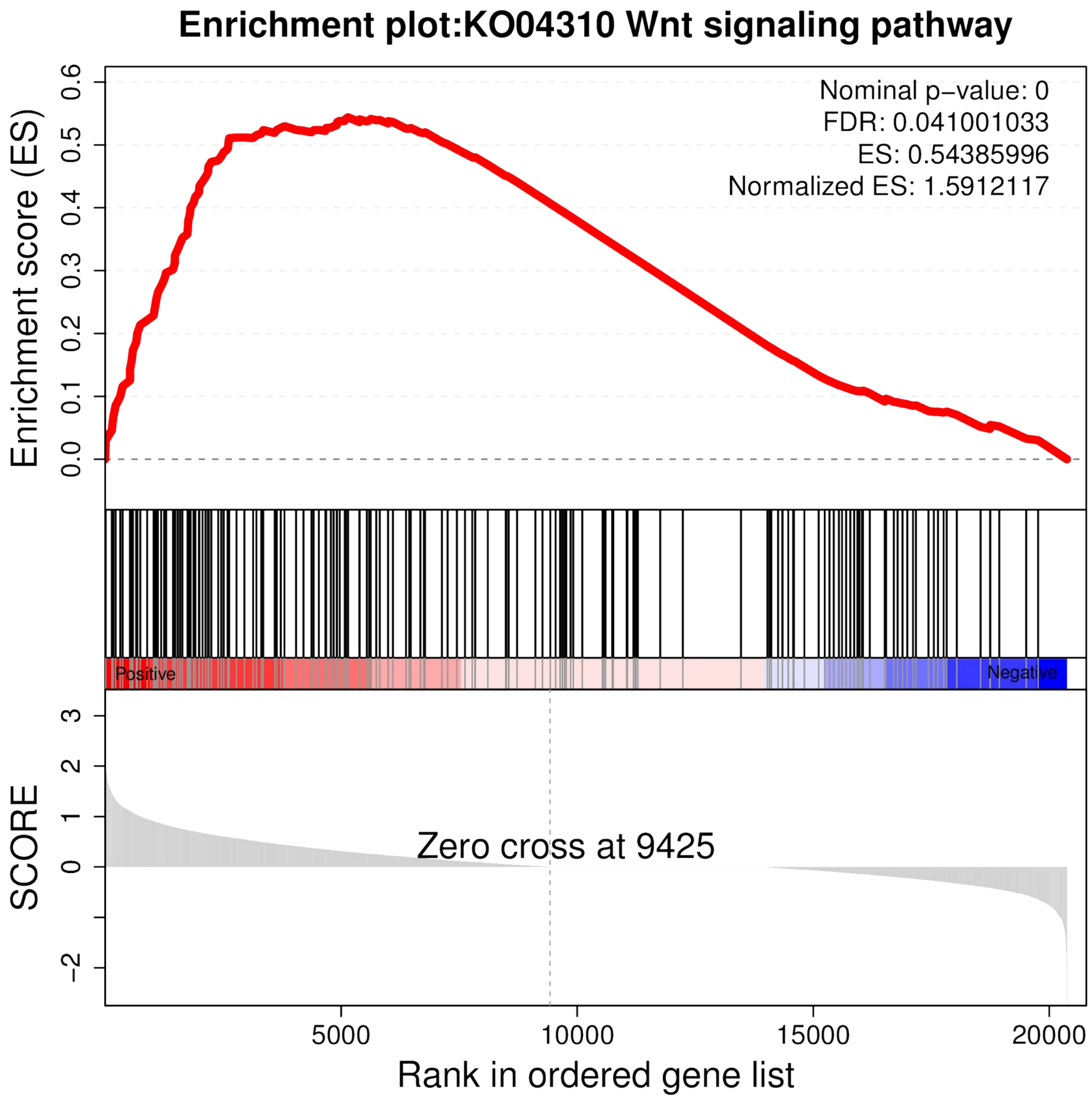

Enrichment plot:KO04310 Wnt signaling pathway
Nominal p−value: 0
FDR: 0.041001033
ES: 0.54385996
Normalized ES: 1.5912117
Enrichment score (ES)
0.0
0.1
0.2
0.3
0.4
0.5
0.6
Positive
Negative
SCORE
Zero cross at 9425
−2
0
1
2
3
5000
10000
15000
20000
Rank in ordered gene list
