# Supplementary material for: Indole‐3‐Propionic Acid Alleviates Metabolic Dysfunction‐Associated Fatty Liver Disease via AHR/AMPK Signaling Activation
Source: Food Sci Nutr. 2026 Aug 7;14(8):e72206. doi: 10.1002/fsn3.72206 (PMC13449033; doi:10.1002/fsn3.72206)
Supplement: Supplementary file 1 [file FSN3-14-e72206-s001.docx]

**Reagent**

**Table S1. Main reagents and manufacturers**

| **Reagent** | **Manufacturer** |
| --- | --- |
| Indole-3-propionic acid (98%) | Shanghai Macklin Biochemical Technology Co., Ltd., Shanghai, China |
| D12450H diet | Research Diets, Inc., New Brunswick, NJ, USA |
| D12450H diet | Research Diets, Inc., New Brunswick, NJ, USA |
| TG assay kit (ELISA) | Jiangsu Meimian Industrial Co., Ltd., Jiangsu, China |
| TC assay kit (ELISA) | Jiangsu Meimian Industrial Co., Ltd., Jiangsu, China |
| TNF-α ELISA kit | Jiangsu Meimian Industrial Co., Ltd., Jiangsu, China |
| IL-1β ELISA kit | Jiangsu Meimian Industrial Co., Ltd., Jiangsu, China |
| Mouse Insulin ELISA Kit | Jiangsu Meimian Industrial Co., Ltd., Jiangsu, China |
| Blood Glucose Monitor | Roche Diagnostics (Shanghai) Co., Ltd |
| Blood Glucose Test Strips | Roche Diagnostics (Shanghai) Co., Ltd. |
| TRIzol reagent | Lanjieke Technology Co., Ltd., Beijing, China |
| 2× Robust SYBR Green qPCR ProMix | Ingeny Biotechnology Co., Ltd., Guangzhou, China |
| reScript III RT ProMix for qPCR | Ingeny Biotechnology Co., Ltd., Guangzhou, China |

| **Reagent** | **Manufacturer** |
| --- | --- |
| RIPA lysis buffer | Beyotime Biotechnology, Shanghai, China |
| BCA protein assay kit | Beyotime Biotechnology, Shanghai, China |
| QuickBlock™ Western primary antibody dilution buffer | Beyotime Biotechnology, Shanghai, China |
| Omni-Easy™ instant protein loading buffer | Shanghai Yazyme Biomedical Technology Co., Ltd., Shanghai, China |
| One-step PAGE gel fast preparation kit | Shanghai Yazyme Biomedical Technology Co., Ltd., Shanghai, China |
| Tris/glycine/SDS running buffer (instant granules) | Shanghai Yazyme Biomedical Technology Co., Ltd., Shanghai, China |
| Transfer buffer (instant granules) | Shanghai Yazyme Biomedical Technology Co., Ltd., Shanghai, China |
| PVDF membrane | Merck Millipore, Burlington, MA, USA |
| Omni-ECL™ ultra-sensitive chemiluminescence detection kit | Shanghai Yazyme Biomedical Technology Co., Ltd., Shanghai, China |
| TBS/Tween buffer (instant granules) | Shanghai Yazyme Biomedical Technology Co., Ltd., Shanghai, China |
| Skim milk powder | Shanghai Yazyme Biomedical Technology Co., Ltd., Shanghai, China |
| Minimum Essential Medium (MEM) | Gibco, Thermo Fisher Scientific, Waltham, MA, USA |
| RPMI 1640 medium | Gibco, Thermo Fisher Scientific, Waltham, MA, USA |

| **Reagent** | **Manufacturer** |
| --- | --- |
| Fetal bovine serum (FBS) | Gibco, Thermo Fisher Scientific, Waltham, MA, USA |
| Trypsin-EDTA solution | Gibco, Thermo Fisher Scientific, Waltham, MA, USA |
| Penicillin-streptomycin(antibiotic-antimycotic) | Gibco, Thermo Fisher Scientific, Waltham, MA, USA |
| Cell Counting Kit-8 (CCK-8) | Biosharp Life Sciences, Hefei, China |
| HepG2 cells | National Collection of Authenticated Cell Cultures, Chinese Academy of Sciences, Shanghai, China |
| Oleic acid (OA) | Shanghai Macklin Biochemical Technology Co., Ltd., Shanghai, China |
| Palmitic acid (PA) | Shanghai Macklin Biochemical Technology Co., Ltd., Shanghai, China |
| Saturated Oil Red O working solution | Solarbio Science & Technology Co., Ltd., Beijing, China |
| Triglyceride (TG) assay kit | Nanjing Jiancheng Bioengineering Institute, Nanjing, China |
| Total cholesterol (TC) assay kit | Nanjing Jiancheng Bioengineering Institute, Nanjing, China |
| AHR antagonist 5 free base | Ambeed, Inc., Arlington Heights, IL, USA |
| AMPK-IN-3 | Ambeed, Inc., Arlington Heights, IL, USA |
| Ampkinone | MedChemExpress (MCE), Monmouth Junction, NJ, USA |

| **Reagent** | **Manufacturer** |
| --- | --- |
| Anti-β-actin antibody | Abcam, Cambridge, UK |
| Goat Anti-Mouse IgG (H+L) HRP | Affinity Biosciences, Ltd., Cincinnati, OH, USA |
| Goat Anti-Rabbit IgG (H+L) HRP | Affinity Biosciences, Ltd., Cincinnati, OH, USA |
| Lamin B1 mouse monoclonal antibody | Affinity Biosciences, Ltd., Cincinnati, OH, USA |
| Aryl hydrocarbon receptor (AhR) antibody | Affinity Biosciences, Ltd., Cincinnati, OH, USA |
| Cytochrome P450 1A1/2 (CYP1A1/2) antibody | Affinity Biosciences, Ltd., Cincinnati, OH, USA |
| Phospho-AMPK alpha(Thr172) antibody | Affinity Biosciences, Ltd., Cincinnati, OH, USA |
| SREBP1 antibody | Affinity Biosciences, Ltd., Cincinnati, OH, USA |
| FAS antibody | Affinity Biosciences, Ltd., Cincinnati, OH, USA |
